# The rise and fall of rationality in language

**DOI:** 10.1073/pnas.2107848118

**Published:** 2021-12-16

**Authors:** Marten Scheffer, Ingrid van de Leemput, Els Weinans, Johan Bollen

**Affiliations:** ^a^Department of Environmental Sciences, Wageningen University, 6700 AA Wageningen, The Netherlands;; ^b^Department of Industrial Engineering and Innovation Sciences, Eindhoven University of Technology, 5600 MB Eindhoven, The Netherlands;; ^c^Department of Informatics, Cognitive Science Program, Indiana University, Bloomington, IN 47408

**Keywords:** language, rationality, sentiment, collectivity, individuality

## Abstract

The surge of post-truth political argumentation suggests that we are living in a special historical period when it comes to the balance between emotion and reasoning. To explore if this is indeed the case, we analyze language in millions of books covering the period from 1850 to 2019 represented in Google nGram data. We show that the use of words associated with rationality, such as “determine” and “conclusion,” rose systematically after 1850, while words related to human experience such as “feel” and “believe” declined. This pattern reversed over the past decades, paralleled by a shift from a collectivistic to an individualistic focus as reflected, among other things, by the ratio of singular to plural pronouns such as “I”/”we” and “he”/”they.” Interpreting this synchronous sea change in book language remains challenging. However, as we show, the nature of this reversal occurs in fiction as well as nonfiction. Moreover, the pattern of change in the ratio between sentiment and rationality flag words since 1850 also occurs in *New York Times* articles, suggesting that it is not an artifact of the book corpora we analyzed. Finally, we show that word trends in books parallel trends in corresponding Google search terms, supporting the idea that changes in book language do in part reflect changes in interest. All in all, our results suggest that over the past decades, there has been a marked shift in public interest from the collective to the individual, and from rationality toward emotion.

## Significance

The post-truth era has taken many by surprise. Here, we use massive language analysis to demonstrate that the rise of fact-free argumentation may perhaps be understood as part of a deeper change. After the year 1850, the use of sentiment-laden words in Google Books declined systematically, while the use of words associated with fact-based argumentation rose steadily. This pattern reversed in the 1980s, and this change accelerated around 2007, when across languages, the frequency of fact-related words dropped while emotion-laden language surged, a trend paralleled by a shift from collectivistic to individualistic language.

The post-truth era where “feelings trump facts” (1) may seem special when it comes to the historical balance between emotion and reasoning. However, quantifying this intuitive notion remains difficult as systematic surveys of public sentiment and worldviews do not have a very long history. We address this gap by systematically analyzing word use in millions of books in English and Spanish covering the period from 1850 to 2019 (2). Reading this amount of text would take a single person millennia, but computational analyses of trends in relative word frequencies may hint at aspects of cultural change (2–4). Print culture is selective and cannot be interpreted as a straightforward reflection of culture in a broader sense (5). Also, the popularity of particular words and phrases in a language can change for many reasons including technological context (e.g., *carriage* or *computer*), and the meaning of some words can change profoundly over time (e.g., *gay*) (6). Nonetheless, across large amounts of words, patterns of change in frequencies may to some degree reflect changes in the way people feel and see the world (2–4), assuming that concepts that are more abundantly referred to in books in part represent concepts that readers at that time were more interested in. Here, we systematically analyze long-term dynamics in the frequency of the 5,000 most used words in English and Spanish (7) in search of indicators of changing world views. We also analyze patterns in fiction and nonfiction separately. Moreover, we compare patterns for selected key words in other languages to gauge the robustness and generalizability of our results. To see if results might be specific to the corpora of book language we used, we analyzed how word use changed in the *New York Times* since 1850. In addition, to probe whether changes in the frequency of words used in books does indeed reflect interest in the corresponding concepts we analyzed how change in Google word searches relates to the recent change in words used in books. Following best-practice guidelines (8) we standardized word frequencies by dividing them by the frequency of the word “an,” which is indicative of total text volume, and subsequently taking z-scores (*SI Appendix*, sections 1, 5, and 8).

## Principal Components of Change

**Fig. 1.**
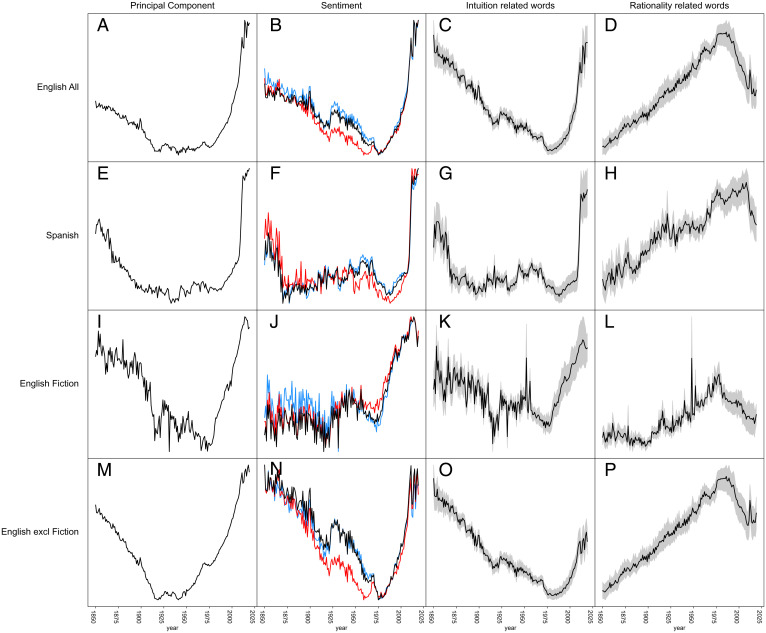
Dynamics of four characteristics of English and Spanish book language represented in Google n-gram data. (*A*, *E*, *I*, and *M*) Second principal component of change in z-scores of frequencies of the 5,000 most-used words. (*B*, *F*, *J*, and *N*) Relative level of arousal (black), positive sentiment (blue), and negative sentiment (red). (*C*, *G*, *K*, and *O*) Z-scores of frequencies of flag-words related to intuition, believing, spirituality, sapience: *spirit*, *imagine*, *wisdom*, *wise*, *hunch*, *mind*, *suspicion*, *believe*, *think*, *trust*, *faith*, *truth*, *true*, *belief*, *doubt*, *hope*, *fear*, *life*, *soul*, *heaven*, *eternal*, *mortal*, *holy*, *god*, *pray*, *mystery*, *sense*, *feel*, *soft*, *hard*, *cold*, *hot*, *smell*, *foul*, *taste*, *sweet*, *bitter*, *hear*, *sound*, *silence*, *loud*, *see*, *light*, *dark*, *bright* (for Spanish: *espíritu*, *imaginar*, *sabiduría*, *mente*, *sospecha*, *creer*, *pensar*, *fe*, *verdad*, *duda*, *esperanza*, *miedo*, *vida*, *alma*, *cielo*, *santo*, *dios*, *misterio*, *sentido*, *sensación*, *sentir*, *suave*, *duro*, *frío*, *caliente*, *gusto*, *dulce*, *oír*, *silencio*, *fuerte*, *ver*, *mirar*, *oscuro*, *brillante*). The black central line represents the mean and the gray shaded area the 95% confidence interval of the mean. (*D*, *H*, *L*, and *P*) Similar but for flag words related to rationality, science, and quantification: *science*, *technology*, *scientific*, *chemistry*, *chemicals*, *physics*, *medicine*, *model*, *method*, *fact*, *data*, *math*, *analysis*, *conclusion*, *limit*, *result*, *determine*, *transmission*, *assuming*, *system*, *size*, *unit*, *pressure*, *area*, *percent* (for Spanish: *ciencia*, *tecnología*, *científico*, *química*, *productos*, *física*, *medicina*, *modelo*, *método*, *dato*, *datos*, *hipótesis*, *estadísticas*, *cálculo*, *análisis*, *conclusión*, *límite*, *resultado*, *determinar*, *transmisión*, *sistema*, *tamaño*, *unidad*, *presión*, *área*, *densidad*, *porcentaje*).

Analyzing language change can imply the risk of cherry-picking. Therefore, as a first unbiased exploration, we perform a principal component analysis (PCA) on the z-scores of relative word frequencies in books over time (*SI Appendix*, section 2). This approach seeks to capture patterns of change in a large dataset without relying on prior assumptions or search images. In both Spanish and English, the first principal component (PC1) corresponds to a monotonic trend over time. The second principal component (PC2) shows an asymmetric U-curve, or “tilted hockeystick,” declining gradually since the industrial revolution and surging sharply in recent decades (Fig. 1, first column). Examination of the words that score highly on opposite ends of either principal component axis (Table 1 and *SI Appendix*, section 10) suggests that in both English and Spanish the monotonic axis captures general trends of word popularity over time. On the high end (representing earlier times) we find more archaic terms such as *civilized*, *ox*, *straw*, *savage*, *carriage*, and *sheriff*. On the low end (corresponding to more recent times) we find words such as *cola*, *product*, *ski*, *allergic*, *tech*, and *dummy*. By contrast, the tilted hockeystick axes are dominated on the high side (more recently) by words reflecting concepts related to personal experience such as senses, spirituality, emotions, and personal relationships as detailed later (Table 1). By contrast, the opposite end of this axis has words related to society including words associated with science, rational decision-making, procedures, and systems (Table 1).

**Table 1.** Contrasting classes of concepts related to a personal (top row) vs. societal view of the world (bottom row) emerge by ranking words according to their correlation with principal components, overall sentiment, and the hockeystick pattern

**Words scoring highest on surging PCA axis (PC2):** angry, look, walk, unexpected, sleep, voice, imagine, embarrassed, tortured, heal, struggling, knowing, potion, ambush, incredible, looking, greedy, terrified, looks, how, torture, learn, anger, invisible, mother, comfortable, drunk, fade, like, brutal, harsh, yourself, pain, sofa, could, dream, distracted, crying, what, thanks, her, eat, walking, shower, helmet, warn, suspected, sense, luckily, smell	**Words correlating most positively to sentiment:** dressed, nights, beating, mad, forget, perfume, wore, delicious, crowd, dinner, took, sister, whispering, saw, hung, next, shut, bad, together, suddenly, slept, beside, thought, away, stood, another, awake, spoke, alive, drank, me, down, broke, dark, blame, inviting, whisper, drown, too, polite, moment, dragged, life, hang, quietly, forgot, glow, silence, footsteps, surprised	**Words declining before 1980 and rising after 1980:** perfect, understood, throw, them, embrace, sight, comfort, nothing, rushing, place, trusting, awful, beautiful, ever, hearts, never, awake, throwing, when, sweet, promise, fallen, threw, cheer, brother, so, spirit, breathe, every, owe, believing, thankful, footsteps, him, rest, stranger, gorgeous, seeing, supposed, ashes, surprised, joy, cheering, disappoint, stood, thrown, dare, who, shine, appetite
**Words scoring lowest on surging PCA axis (PC2):** secretary, state, report, year, sec, council, order, authorized, district, west, eastern, behalf, northern, president, office, statement, under, January, vice, attorney, east, committee, resident, October, south, reference, officer, branch, annual, interest, prepared, following, commonwealth, August, counsel, exclusive, further, board, April, collected, November, February, July, jersey, September, jurisdiction, general, contract, permanent, remaining	**Words correlating most negatively to sentiment:** deputy, separate, annual, surface, applied, report, joint, contain, sub, marine, effect, determined, counsel, established, foreign, reasonable, congress, qualified, gross, number, direct, violation, assigned, tables, increase, request, section, savings, remaining, temperature, library, permit, construction, funds, reference, chemistry, transportation, manual, provided, volume, capital, chemical, assist, public, member, retarded, demonstration, affected, department, rate	**Words rising before 1980 and declining after 1980:** area, program, indicate, available, development, basis, determine, initial, technical, million, addition, final, range, replacement, personnel, control, unit, involved, percent, eliminate, limited, rate, concentration, increase, result, test, staff, included, tested, transfer, maximum, zone, plus, sample, recent, congressman, level, funds, data, responsible, basic, laboratory, equipment, budget, procedure, breakdown, effective, activity, tape, review

Listed are the words that score highest vs. lowest on the second PCA axis depicted in Fig. 1, the words that correlate most positively vs. negatively with positive sentiment, and the words that increased most clearly after 1980 while declining between 1850 and 1980 vs. words that show the opposite pattern (ranked to the absolute difference in Kendall tau in those periods). We used positive sentiment for computing the correlations in the second column, but this is closely correlated to negative sentiment and arousal. Longer lists (5%) of English, and the analogous analysis of Spanish words, English fiction, and English excluding fiction are presented in *SI Appendix*, section 10.

## Sentiment Trends

As a next objective step, we analyzed changes in the relative frequencies of words that have been independently assessed as indicators of different aspects of emotion (using the ANEW [Affective Norms for English Words] lexicon) ( 9) and a comparable lexicon for Spanish (see *SI Appendix*, section 3). “Valence” or pleasantness associated with a word is a dominant aspect of many models of human emotion. ANEW valence values range from low (e.g., for words such as *torture*, *rape*, *terrorism*) to high (e.g., words such as *vacation*, *enjoyment*, *free*). Some models of human emotions also include an orthogonal affective dimension of arousal, which can be evoked by words going from low arousal (e.g., *dull*, *scene*, *asleep*) to high arousal (e.g., *rampage*, *sex*, *tornado*). Integrating frequency-weighted valence and arousal sentiment scores for all words from our set of 5,000 that were listed in the sentiment lexicons we arrive at an index of positive and negative sentiment as well as arousal for the entire content of books published each year since 1850. In both English and Spanish we find patterns of these affective indicators that closely resemble the hockeystick patterns of the PCA (Fig. 1, second column and *SI Appendix*, Table S1).

## Correlated Concepts

**Fig. 2.**
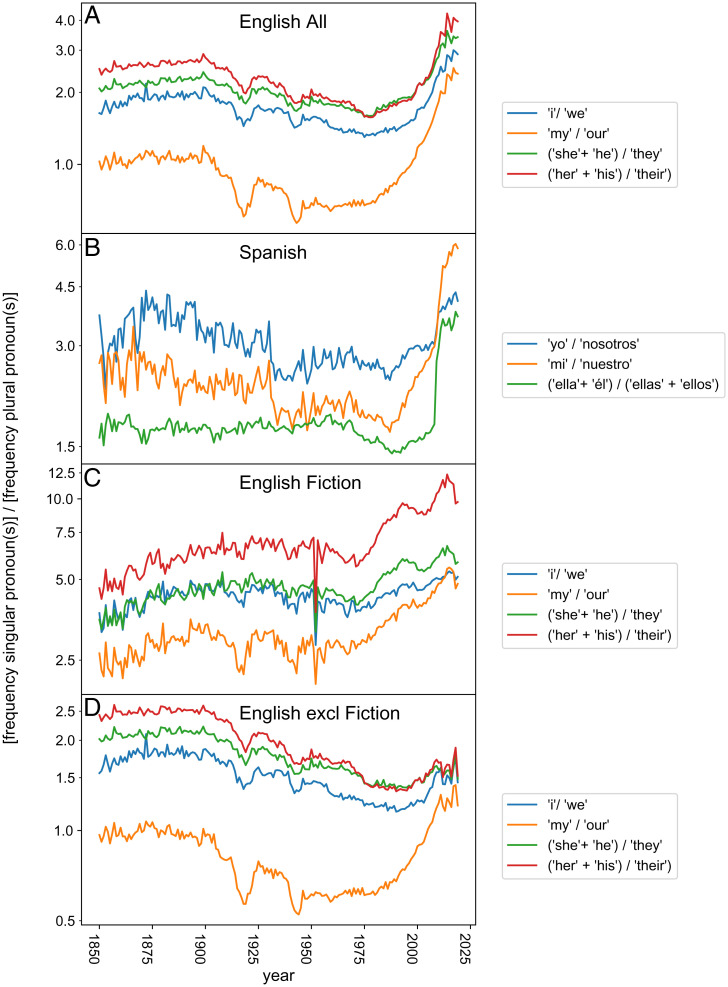
(*A*–*D*) Ratio of the relative frequencies of singular to corresponding plural pronouns in various book corpora represented in the Google n-gram database.

Although sentiment analysis allows a meaningful evaluation of the affective components of language change, sentiment indicators do not necessarily capture the full essence of the sea change revealed by the PCA. This becomes evident if we examine the words correlating and anticorrelating most strongly to the PCA axis, to the sentiment score, and to the overall hockeystick pattern. Lists of the 5% top-scoring words are given in *SI Appendix*, section 10), but a glance at the 1% top-scoring words for English already reveals that the patterns we find correspond to a seesaw between two opposing poles of concepts (Table 1). On the one hand we have words that may be broadly characterized as related to a personal view of the world (Table 1, top row). At the contrasting end there are words that could be characterized at first glance as related to societal systems (Table 1, bottom row). The idea that there has been a shift in emphasis from the collective to the individual over the past decades is supported by a pronounced trend toward the use of singular versus plural pronouns starting in the 1980s (Fig. 2).

A closer look at the short and also the longer (*SI Appendix*) lists of words suggests that both the personal and the societal poles encompass several distinct groups of concepts. On the personal pole we have words that we could classify as related to belief, spirituality, sapience, and intuition (e.g., *imagine*, *compassion*, *forgiveness*, *heal*), senses (e.g., *feel*, *smell*, *silence*), and the body (e.g., *knee*, *face*, *chest*) but also personal pronouns (e.g., *me*, *you*, *she*) and activities (e.g., *walk*, *sleep*, *smile*). By contrast, at the opposing pole tentatively labeled as societal we have words related to science and technology (e.g., *experiment*, *circuit*, *chemistry*, *dean*, *gravity*), quantification (e.g., *weigh*, *depth*, *greater*, *per*), business and economy (e.g., *corporation*, *commissioner*, *salary*, *cost*, *shipping*, *contract*), social organization (e.g., *jurisdiction*, *congress*, *minister*, *department*, *commission*, *institution*), and time and place (e.g., *year*, *month*, *january*, *district*, *west*).

To test if those tentatively discerned concepts do indeed contribute individually to the seesaw that we see in the PCA and sentiment, we examined the historical dynamics of the separate concept groups. Most of the groups are straightforward to delineate. To obtain suggestions for populating the belief, spirituality, and intuition cluster we also used a thesaurus algorithm available at relatedwords.org (combining search techniques such as word embedding and Concept-Net). Examining dynamics of each of the resulting clusters reveals a striking synchrony, confirming that within each language the shifts in interest across the concepts we identified happen very much in concert (*SI Appendix*, section 4).

**Fig. 3.**
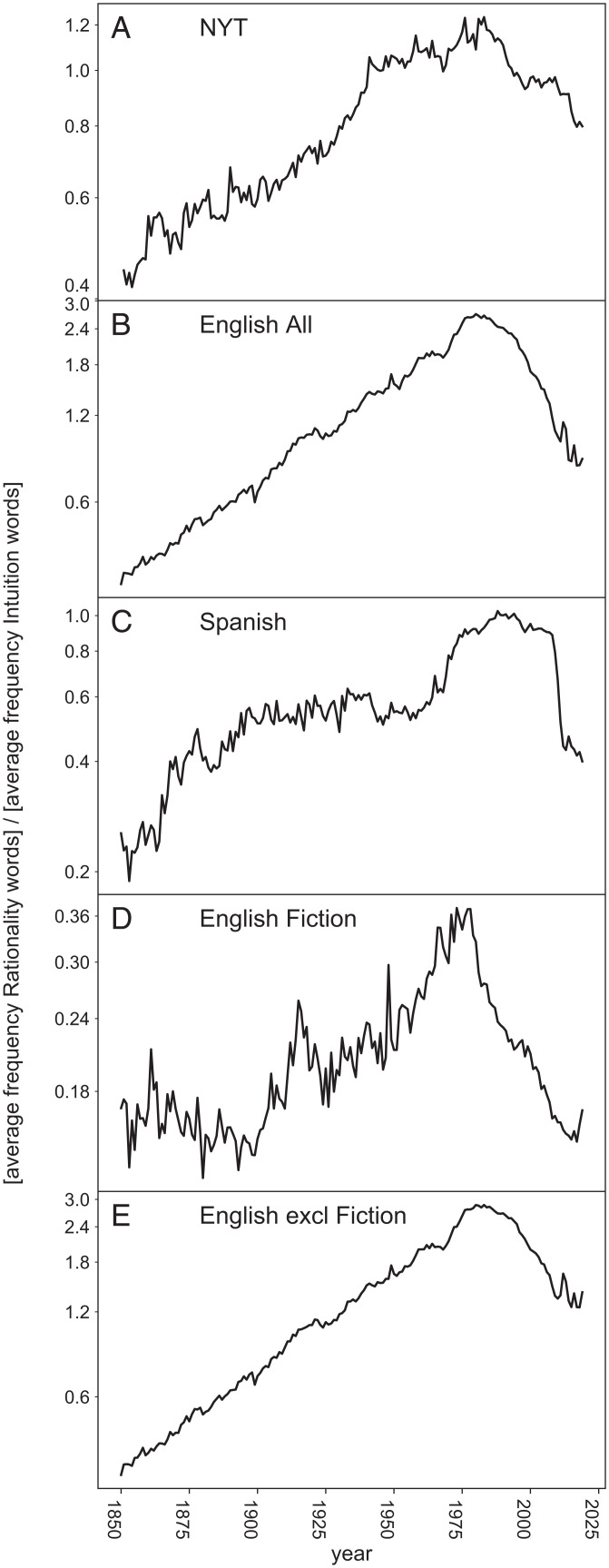
Ratio of intuition to rationality related words in the *New York Times* (*A*) and various book corpora represented in the Google n-gram database (*B*–*E*). The graphs depict the ratio of the mean relative frequencies of the sets of rationality-related and intuition-related flag words presented in Fig. 1, right-hand columns.

Arguably, the opposing poles of human versus societal concepts we identified may also be interpreted in terms of how they relate to two fundamentally different cognitive modes of operation (10–13), namely system I (“thinking fast,” loosely intuition) vs. system II (“thinking slow,” loosely rationality). We test this idea by exploring clusters of words that we now filter to specifically reflect those opposed modes of thinking (for details see *SI Appendix*, section 4). Selected intuition flag words are rooted in the concepts of belief, spirituality, sapience, intuition, and senses, while the rationality flag words we used are rooted in the concepts of science, technology, and quantification (see *SI Appendix*, section 4 for a full account). Plotting the dynamics of the frequencies of words in those clusters supports the view that the PCA and sentiment patterns we revealed are correlated to a systematic change along the rationality–intuition gradient (Figs. 1 and 3). Moreover, looking at a small set of intuition and rationality flag words across a larger collection of languages (American English, British English, German, French, Italian, and Russian) we find roughly similar patterns (*SI Appendix*, section 9).

## Fiction vs. Nonfiction

The Google n-gram corpus has an English Fiction category, which is a subset from the overall English corpus, thus making it possible to estimate word frequencies in the English subset excluding fiction too (see *SI Appendix*, section 6). While the resulting corpus (English excluding Fiction) cannot be considered free of fiction, it may safely be assumed that its relative proportion of nonfiction is substantially higher than that in the fiction corpus. Analysis of those subsets reveals that the proportion of fiction in the n-gram database rose from about 5% up till 1975 to about 35% in recent years (see *SI Appendix*, Fig. S9). To explore how this affects the word balance in the overall corpus we analyzed the patterns separately for English fiction and nonfiction (Figs. 1–3 and *SI Appendix*, section 6). It turns out that the dynamics of intuition flag words and rationality-related words run in close parallel in fiction and nonfiction. The magnitude of the surge in sentiment- and intuition-related words is stronger in the overall English corpus than in the nonfiction corpus (Fig. 1 *B* and *C* vs. *N* and *O*). This is likely an effect of rising proportion of fiction in the database over the past decades, as fiction is more biased towards intuition-related words (compare the rationality–intuition ratio in Fig. 3 *D* and *E*). More generally, this result illustrates how changes in the representation of contrasting genres in the Google n-gram database can affect relative frequencies of words that are overly abundant or rare in particular genres. As Google’s policy for including books has changed over the past decade we also analyzed the 2009 version of the n-gram database. Patterns turn out to remain robust, even though the relative proportion of genres in this older database was different (see *SI Appendix*, section 7). Taken together these results suggest that changes in the relative interest in the dimensions of language we probed tend to be reflected rather broadly across genres.

## Comparison with *New York Times* and Google Search Queries

**Fig. 4.**
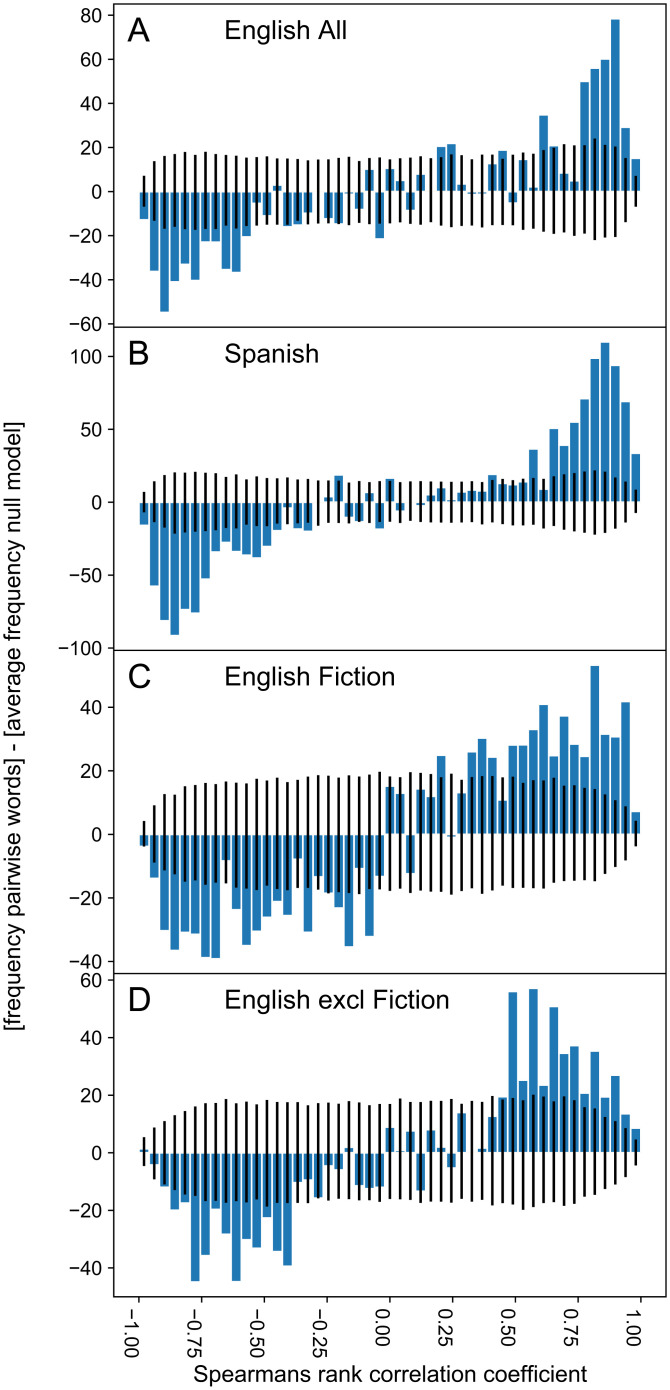
(*A*–*D*) Relationship between trends in the use of words in Google search queries and use of the same words in books for the period 2004 to 2019. Blue bars represent frequency distributions of Spearman rank correlations between each word in books and that same word in Google searches after subtracting average frequencies predicted by a null model of randomly matched words (50 bins). To construct the null model we matched the 5,000 words in books with randomly picked words from the same word list in Google searches and calculated the Spearman rank correlation. We ran this 1,000 times, resulting in a frequency distribution for each correlation bin. We subtracted the mean from the resulting distribution, such that the null line represents the average frequency of correlations (for more details see *SI Appendix*). Black lines represent the 5% and 95% percentiles of the frequency distributions of the null model per correlation bin. For each of the corpora, positive correlations are found more than expected by chance, while negative correlations are found less than expected.

For comparison with the book analyses we retrieved the use of the two groups of key words (the same as shown in Fig. 1, right-hand columns) in the *New York Times* since 1850 (see *SI Appendix*, section 5). The fraction of articles in which any of those words occurs tends to rise over time (*SI Appendix*, section 5). However, the balance between rational and intuition words reflects the same overall trend we find for books (Fig. 3), suggesting that neither the long-term pattern nor its recent reversal are specific to the book corpus. When it comes to interpretation, an important question that remains is whether trends in word frequencies do to some extent reflect trends in public interest in the corresponding concepts. This is difficult to probe in a quantitative way. Nonetheless, since 2004 one proxy for interest is the frequency with which people search a word using Google. We therefore compared the trends in book words from 2004 till 2019 with trends of the same words in the same time period in Google search queries (from https://trends.google.com; see *SI Appendix*, section 8). It turns out that change in interest for particular words in Google search queries is more often positively (and less often negatively) correlated to trends in use of the same words in books than expected by chance (Fig. 4). This suggests that indeed trends in book language do in part reflect trends in interest.

## Robustness and Potential Biases

Despite the close relationship between book trends and Google search interests in recent years, it remains possible that the long-term patterns we find are in part artifacts of the data and our choice of words. With respect to the latter, the 5,000 most frequent words in any language represent an overwhelming sample of common language use, buffering against the problem that any individual word may be subject to fashions or change meaning. Our findings may, however, be subject to other systematic biases. For instance, our list of 5,000 most common words and the emotional ranking of words was determined in recent years and therefore reflects relatively recent language use. Still, probably the most important caveat of using book texts is that they are a biased representation of language, a bias that may change over time (14, 15). What ends up in the university libraries used for the Google n-gram data varies with trends in Google’s book-inclusion policy, editorial practices, library policies, and popularity of genres. As none of those effects can be excluded it is important that we find the same trends for word use in the *New York Times*. Also, the observation that rationality- and intuition-related words have the same trends in fiction and in the general corpus minus assigned fiction suggests that underlying shifts in attitude toward those opposite poles of thinking tend to be reflected across genres, an assertion consistent with the finding that legislative texts have also become more informal over the past decades (16).

It is also worth noting that the link between book language and social sentiment has been validated in other studies (17) and that the long-term trend we find until 1980 is in line with what has been found in other studies including different text corpora and different indicators. For instance, a study comparing a corpus of *New York Times* articles from 1851 to 2015 and the Google Books corpus from 1800 to 2000 showed (18) that in both corpora there has been a significant downward trend in positive as well as negative words as classified through the Linguistic Inquiry and Word Count (LIWS) system (19). Another study, using a corpus from the *New York Times*, a corpus of scientific articles, and a corpus of Google Books, revealed that over the past two centuries in all those corpora there has been a significant increase of words reflecting causal reasoning as reflected by words in the “cause” category in the LIWS system (a list of 108 words such as *because*, *since*, *hence*, *how*, *why*, *depends*, and *implies*) (20).

In conclusion, we cannot exclude the possibility that our results are biased by as-yet-unknown effects in the data. For instance, the rise of more casual language in the Google Books data could be related to the digital transformation enabling libraries to collect a larger diversity of materials while staying within budget. Such “known unknowns” as well as yet “unknown unknowns” should be subject to future research. Nonetheless, studies using different corpora as well as different marker words confirm the long-term decline of sentiment-laden (positive and negative) language and a rise of words related to causal reasoning. Meanwhile, the dramatic recent reversal of this trend occurs in fiction as well as a corpus from which much fiction has been filtered and is also found in our *New York Times* analysis. Thus, while it will be important to explore alternative word collections, sentiment classifications, and text corpora it seems likely that the marked U-shaped pattern we find reflects a true dimension of language change.

## Potential Drivers

Inferring the drivers of this stark pattern necessarily remains speculative, as language is affected by many overlapping social and cultural changes. Nonetheless, it is tempting to reflect on a few potential mechanisms. One possibility when it comes to the trends from 1850 to 1980 is that the rapid developments in science and technology and their socioeconomic benefits drove a rise in status of the scientific approach, which gradually permeated culture, society, and its institutions ranging from the education to politics. As argued early on by Max Weber, this may have led to a process of “disenchantment” as the role of spiritualism dwindled in modernized, bureaucratic, and secularized societies (21, 22).

What precisely caused the observed stagnation in the long-term trend around 1980 remains perhaps even more difficult to pinpoint. The late 1980s witnessed the start of the internet and its growing role in society. Perhaps more importantly, there could be a connection to tensions arising from neoliberal policies which were defended on rational arguments, while the economic fruits were reaped by an increasingly small fraction of societies (23–25).

In many languages the trends in sentiment- and intuition-related words accelerate around 2007 (*SI Appendix*, section 9). One possible explanation could be that the standards for inclusion in Google Books shifted from “being in a library Google had an agreement with” to “from a publisher that directly deposited with Google” after 2004 to 2007, thus affecting the corpus composition. The 2007 shift also coincides with the global financial crisis which may have had an impact. However, earlier economic crises such as the Great Depression (1929 to 1939) did not leave discernable marks on our indicators of book language. Perhaps significantly, 2007 was also roughly the start of a near-universal global surge of social media. This may be illustrated by plotting the dynamics of the word “Facebook” as a marker alongside the frequency of a set of intuition and rationality flag words in different languages (*SI Appendix*, section 9).

Various lines of evidence underpin the plausibility of an impact of social media on emotions, interests, and worldviews. For instance, there may be negative effects of the use of social media on subjective well-being (26). This can in part be related to distortions such as the perception that your friends are more successful, have more friends, and are happier (27, 28) and more beautiful (29) than you are. At the same time, a perception that problems abound may have been fed by activist groups seeking to muster support (30) and lifestyle movements seeking to inspire alternative choices (31). For instance, social media catalyzed the Arab Spring, among other things, by depicting atrocities of the regime (32), jihadist videos motivate terrorists by showing gruesome acts committed by US soldiers (33), and veganism is promoted by social media campaigns highlighting appalling animal welfare issues (31). Many of the problems highlighted on social media will be real, and they may have been hidden from the public eye in the past. However, independently of whether problems are exaggerated or merely revealed online, the popular effect of such awareness campaigns may be the perception of an unfair world entangled in a multiplicity of crises. Further down the gradient from revelation to exaggeration we find misinformation. The spread of misinformation (34) and conspiracy theories (35) may be amplified by social media, as the online diffusion of false news is significantly broader, faster, and deeper than that of true news and efforts to debunk (36). Conspiracy theories originate particularly in times of uncertainty and crisis (35, 37) and generally depict established institutions as hiding the truth and sustaining an unfair situation (38). As a result, they may find fertile grounds on social media platforms promulgating a sense of unfairness, subsequently feeding antisystem sentiments. Neither conspiracy theories nor the exaggerated visibility of the successful nor the overexposure of societal problems are new phenomena. However, social media may have boosted societal arousal and sentiment, potentially stimulating an antisystem backlash, including its perceived emphasis on rationality and institutions.

Importantly, the trend reversal we find has its origins decades before the rise of social media, suggesting that while social media may have been an amplifier other factors must have driven the stagnation of the long-term rise of rationality around 1975 to 1980 and triggered its reversal. Perhaps a feeling that the world is run in an unfair way started to emerge in the late 1970s when results of neoliberal policies became clear (23–25) and became amplified with the rise of the internet and especially social media. A central role of discontent would be consistent with the rise in language characteristic of so-called cognitive distortions (39) known in psychology as overly negative attitudes toward oneself, the world, and the future (40–43). If disillusion with “the system” is indeed the core driver, a loss of interest in the rationality that helped build and defend the system could perhaps be collateral damage.

## Outlook

It seems unlikely that we will ever be able to accurately quantify the role of different mechanisms driving language change. However, the universal and robust shift that we observe does suggest a historical rearrangement of the balance between collectivism and individualism and—inextricably linked—between the rational and the emotional or framed otherwise. As the market for books, the content of the *New York Times*, and Google search queries must somehow reflect interest of the public, it seems plausible that the change we find is indeed linked to a change in interest, but does this indeed correspond to a profound change in attitudes and thinking? Clearly, the surge of post-truth discourse does suggest such a shift (44–48), and our results are consistent with the interpretation that the post-truth phenomenon is linked to a historical seesaw in the balance between our two fundamental modes of thinking. If true, it may well be impossible to reverse the sea change we signal. Instead, societies may need to find a new balance, explicitly recognizing the importance of intuition and emotion, while at the same time making best use of the much needed power of rationality and science to deal with topics in their full complexity. Striking this balance right is urgent as rational, fact-based approaches may well be essential for maintaining functional democracies and addressing global challenges such as global warming, poverty, and the loss of nature.

## Supplementary Material

Supplementary File

## Data Availability

All codes and data are available at https://github.com/jbollen/rise_and_fall_of_rationality_in_language.

## Change History

January 22, 2022: The byline has been updated.
