# Supplementary material for: The rise and fall of rationality in language
Source: Proc Natl Acad Sci U S A. 2021 Dec 16;118(51):e2107848118. doi: 10.1073/pnas.2107848118 (PMC8713757; doi:10.1073/pnas.2107848118)
Supplement: Supplementary File [file pnas.2107848118.sapp.pdf]

Supplementary material to

The rise and fall of rationality in language

Marten Scheffer, Ingrid van de Leemput, Els Weinans, Johan Bollen

Contents

1. Google books 2019 data and preprocessing 2
2. Principal Component Analysis 2
3. Computing sentiment scores 4
4. Flag-word selection and dynamics in English and Spanish in Google ngrams 4
5. New York Times data 11
6. Fiction and non-fiction 14
7. Google books 2009 data 19
8. Google trends data 23
9. Google books trends in other languages 23
10. Top 5% words correlated to PCA, sentiment, and U-pattern 26

References 40

1. Google books 2019 data and preprocessing

We used the 2019 release of the Google Books n-gram data which the Google Books team made freely available (https://storage.googleapis.com/books/ngrams/books/datasetsv3.html). The data covers a period from the 16th century to the year 2019. Time series from 1850 till 2019 of the 5000 most frequent words in English [1] and Spanish (https://corpus.rae.es/frec/5000_formas.TXT and https://corpus.rae.es/lfrecuencias.html) were extracted from the 1-grams. The word 'war' (Spanish: 'guerra') was removed. The sentiment of 'war' is 2.08 for ANEW (on the original scale from 0 to 10) and the word has a massive increase in frequency around the two world wars. Therefore, it would skew the sentiment analysis around war periods.

To correct for the increasing volume of text towards more recent times, we scaled the time series dividing word frequencies by the frequency of the word 'an' (Spanish: 'la') resulting in relative word frequencies. Our resuls are robust against other indicators of total text volume, such as the frequency of the word 'the', and the mean frequency of all 5000 words. We finally centered and scaled the relative frequencies to their standard deviation. The resulting z-scores were used to perform all analyses.

2. Principal Component Analysis

A PCA finds a direction in a multidimensional space where the variance is maximized, i.e. where most dynamics are observed. The multidimensional space in our case consists of axes that describe the frequency of a word, so every axis reflects the dynamics of one word in our analysis. The obtained coefficients of the principal components reflect the weight that a word has on that particular principal component. The 5% words with the highest coefficient and 5% words with the lowest coefficient (see section 10) were investigated by eye. The complete time series were projected on the first two principal components in order to evaluate the dynamics of the language as a whole in the direction of these PC's.

Words scoring strongly in the two opposite directions on either axes are given in section 10. Examining those words suggests that the first axis in both English and Spanish capture mostly the natural turnover of vocabulary while the second axis in each language is dominated on the high side by words reflecting concepts related to personal experience, while the opposite end is populated by concepts related to society. This 'personal vs society' axis invariably surges in recent decades and is correlated to the sentiment value of words (Table S1).

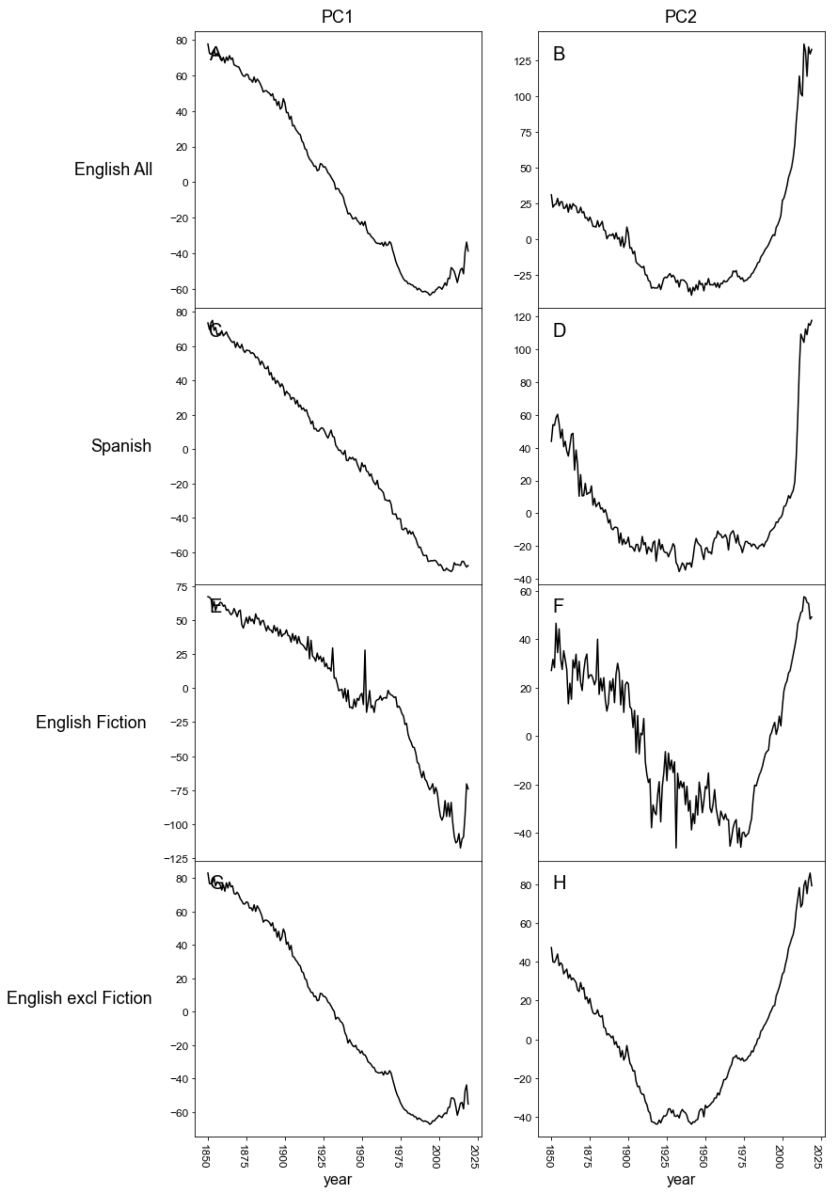

Figure S1. *Time development of the 5000 most used words in English and Spanish represented by the weight on their first two principal components. Note that in each corpus PC2 correlates strongly to sentiment scores and surges in recent decades. This is the axis which we examine further in the main text. Explained variance for English, Spanish, English Fiction, and English excl Fiction resp. are 0.45, 0.44, 0.52, and 0.5 for PC1 and 0.30, 0.23, 0.16, and 0.24 for PC2.*

3. Computing sentiment scores

For each of the words we assigned scores for mean valence and arousal if such scores were available in the sentiment lists that we used for English [2], and Spanish [4]. Since we aimed to separate negative and positive sentiment in our analysis, we first subtracted 5 from the ANEW valence scores, to obtain scores from -5 to 5, where all scores lower than zero reflected negative sentiment whereas all scores higher than zero reflected positive sentiment. Next, we multiplied the z-scores of the relative frequencies with their sentiment score, and arousal score. The sentiment and arousal levels per word for each year were summed over all words, resulting in a sentiment per year.

Table S1. Correlations of positive and negative sentiment with PC2, the PC axis that shows an inverse U curve.

	Positive sentiment		Negative sentiment	
	Spearman correlation coefficient	p-value	Spearman correlation coefficient	p-value
English	0.75*	$9e^{-32}$	0.85*	$3e^{-48}$
Spanish	0.38*	$3e^{-7}$	0.44*	$2e^{-9}$
English Fiction	0.55*	$7e^{-15}$	0.10	0.19
English excl Fiction	0.50*	$3e^{-12}$	0.62*	$3e^{-19}$

4. Flag-word selection and dynamics in English and Spanish in Google ngrams

Our exploration of words that scored strongly on the hockeystick PCA axis or correlated strongly with sentiment led us to tentatively define groups of correlated concepts that seemed to go hand-in-hand. To check for each of those concepts if they indeed follow similar dynamics, we first populated sets of flag-words related to each of the concepts using a thesaurus algorithm available at relatedwords.org (combining search techniques such as word embedding and Concept-Net). Subsequently we plotted dynamics of each group of flag-words separately.
As we are using the 5000-word most frequent word collections for plotting, not all of the words resulting from the thesaurus search end up in the final selections. Also, inevitably, there is no precise 1-to-1 connection between words in the different languages.

Personal related word categories

English:

1) Belief : *spirit, imagine, wisdom, wise, hunch, mind, suspicion, believe, think, trust, faith, truth, true, belief, doubt, hope, fear, life, soul, heaven, eternal, mortal, holy, god, pray, mystery*
2) Senses: *sense, sensation, feel, soft, hard, cold, hot, smell, foul, taste, sweet, bitter, hear, sound, silence, loud, see, gaze, glimpse, light, dark, bright*
3) Body: *head, nose, mouth, ear, hair, shoulder, chest, belly, leg, hand, arm, toe, finger*
4) Personal pronouns*: i, we, you, he, she, they, mine, your, her, his, our, their*
5) Activities: *walk, sleep, shout, sit, laugh, eat, drink, smile*

Spanish:

1) Belief: *espíritu, imaginar, sabiduría, sabio, corazonada, mente, sospecha, creer, pensar, confiar, fe, verdad, creencia, duda, esperanza, miedo, vida, alma, cielo, eterno, mortal, santo, dios, orar, sobrenatural, misterio, sabio*
2) Senses: *sentido, sensación, sentir, suave, duro, frío, caliente, oler, asqueroso, gusto, dulce, amargo, oír, sonar, silencio, fuerte, ver, mirar, vislumbrar, ligero, oscuro, brillante*
3) Body: *cabeza, nariz, boca, oreja, cabello, hombro, pecho, vientre, pierna, mano, brazo, dedo*
4) Personal pronouns: *yo, nosotros, tú, él, ella, ellos, mío, tu, nuestro, su*
5) Activities: *caminar, dormir, gritar, sentarse, reír, comer, beber, sonreír*

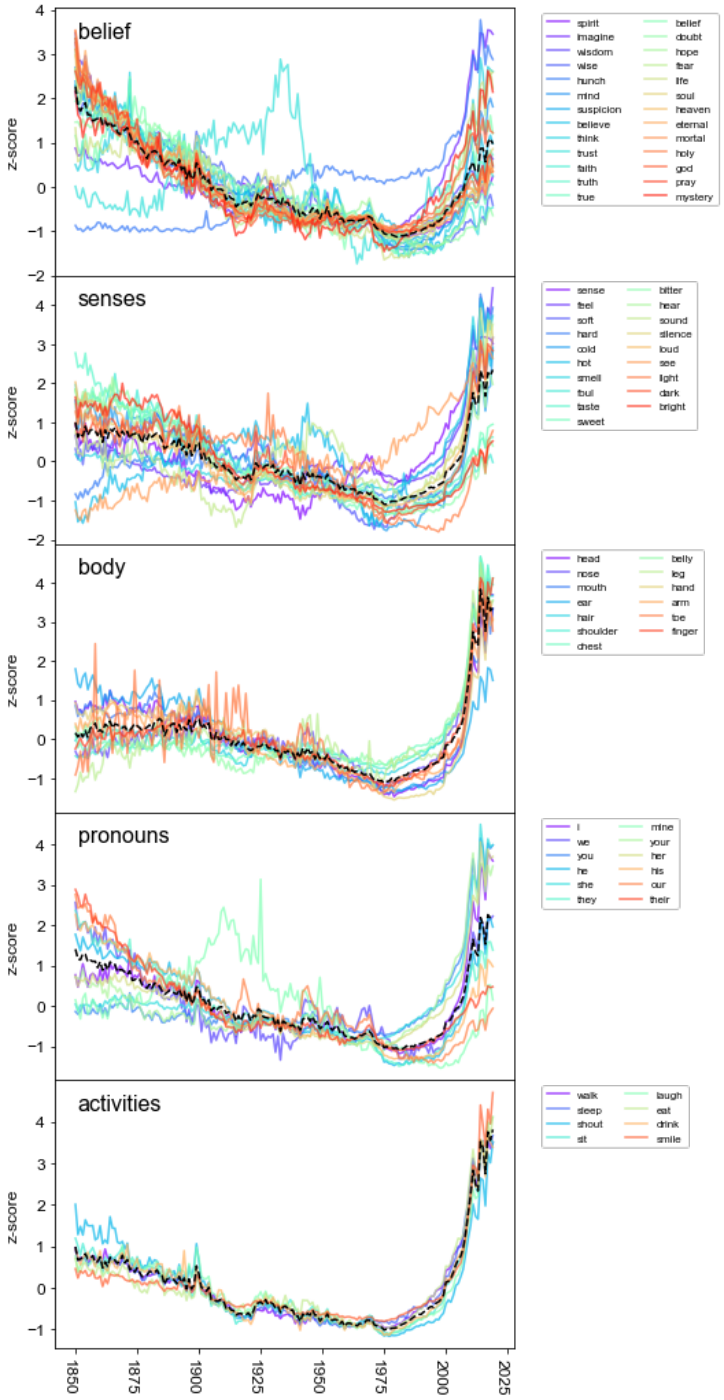

Figure S2. Time development of the frequencies of separate groups of English personal-related flag-words (see text above) in Google ngrams. The central black line represents the mean.

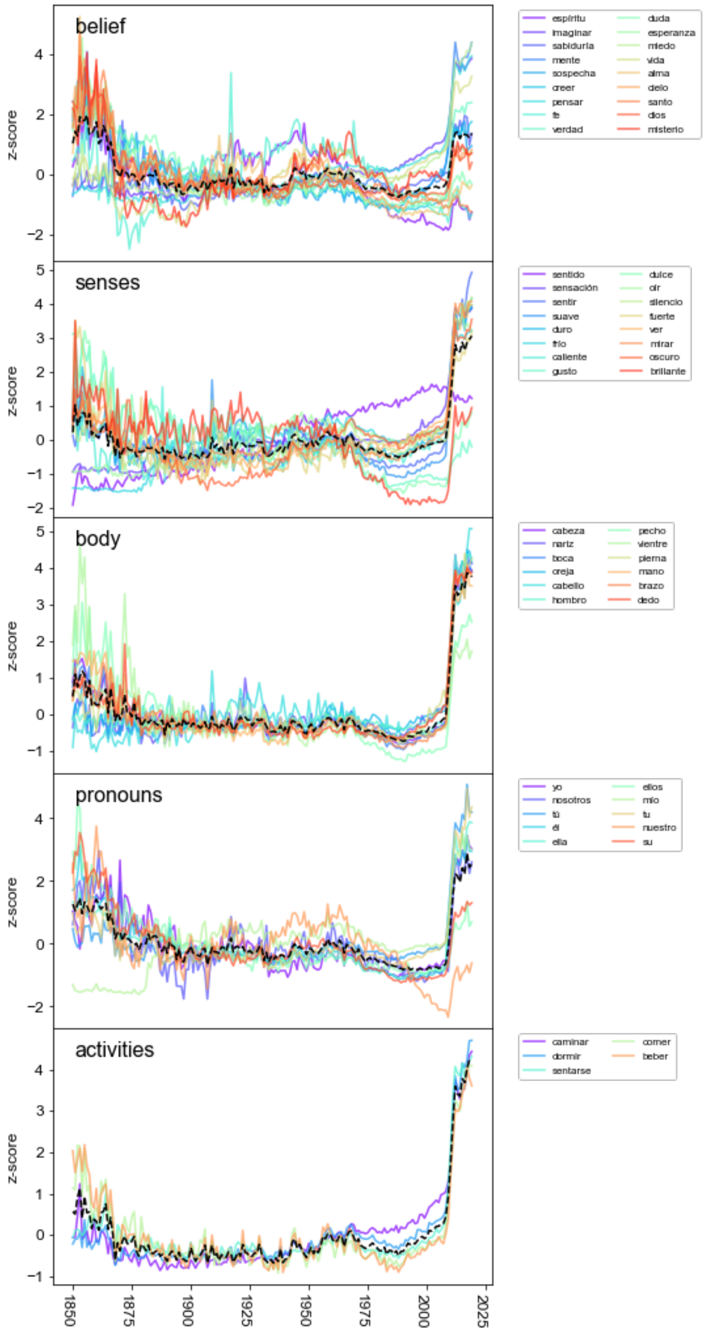

Figure S3. Time development of the frequencies of separate groups of Spanish personal-related flag-words (see text above) in Google ngrams. The central black line represents the mean.

Society related word categories

English

1) Science and technology: *science, technology, scientific, chemistry, chemicals, physics, medicine, uncertainty, model, methodology, method, fact, data, hypothesis, statistics, math, analysis, conclusion, replication, limit, result, determine, transmission, assuming, system*
2) Quantification: *size, unit, pressure, area, density, percent*
3) Business and economy: *business, company, payment, expenses, manager, profit, investment, labour, revenue, market, employee*
4) Social organization: *ministry, commission, lawyer, government, law, nation, community, administration, municipality, education, city, agreement, health, policy, central*
5) Time and place: *year, month, week, january, july, december, monday, friday, country, state, city*

Spanish:

1) Science and technology: *ciencia, tecnología, científico, química, productos, químicos, física, medicina, inseguridad, modelo, metodología, método, dato, datos, hipótesis, estadísticas, cálculo, análisis, conclusión, replicación, límite, resultado, determinar, transmisión, asumiendo, sistema*
2) Quantification: *tamaño, unidad, presión, área, densidad, porcentaje*
3) Business and economy: *comercio, empresa, pago, gastos, gerente, beneficio, inversión, trabajo, ingresos, mercado, trabajadores*
4) Social organization: *ministerio, comité, jurista, gobierno, ley, nación, comunidad, administración, municipio, educación, ciudad, acuerdo, salud, política, central*
5) Time and place: *año, mes, semana, enero, julio, diciembre, lunes, viernes, país, ciudad, estado*

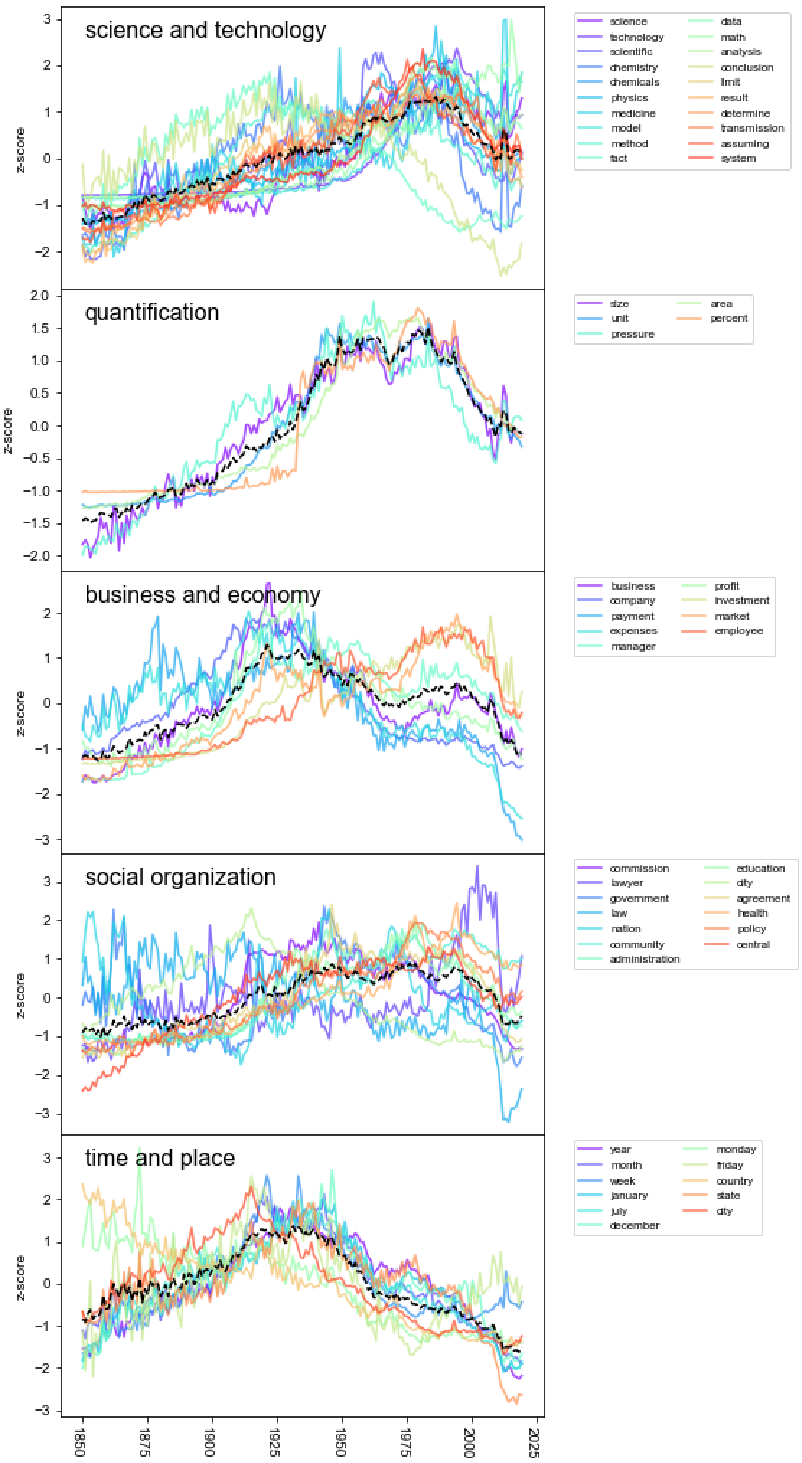

Figure S4. Time development of the frequencies of separate groups of English society-related flag-words (see text above) in Google ngrams. The central black line represents the mean.

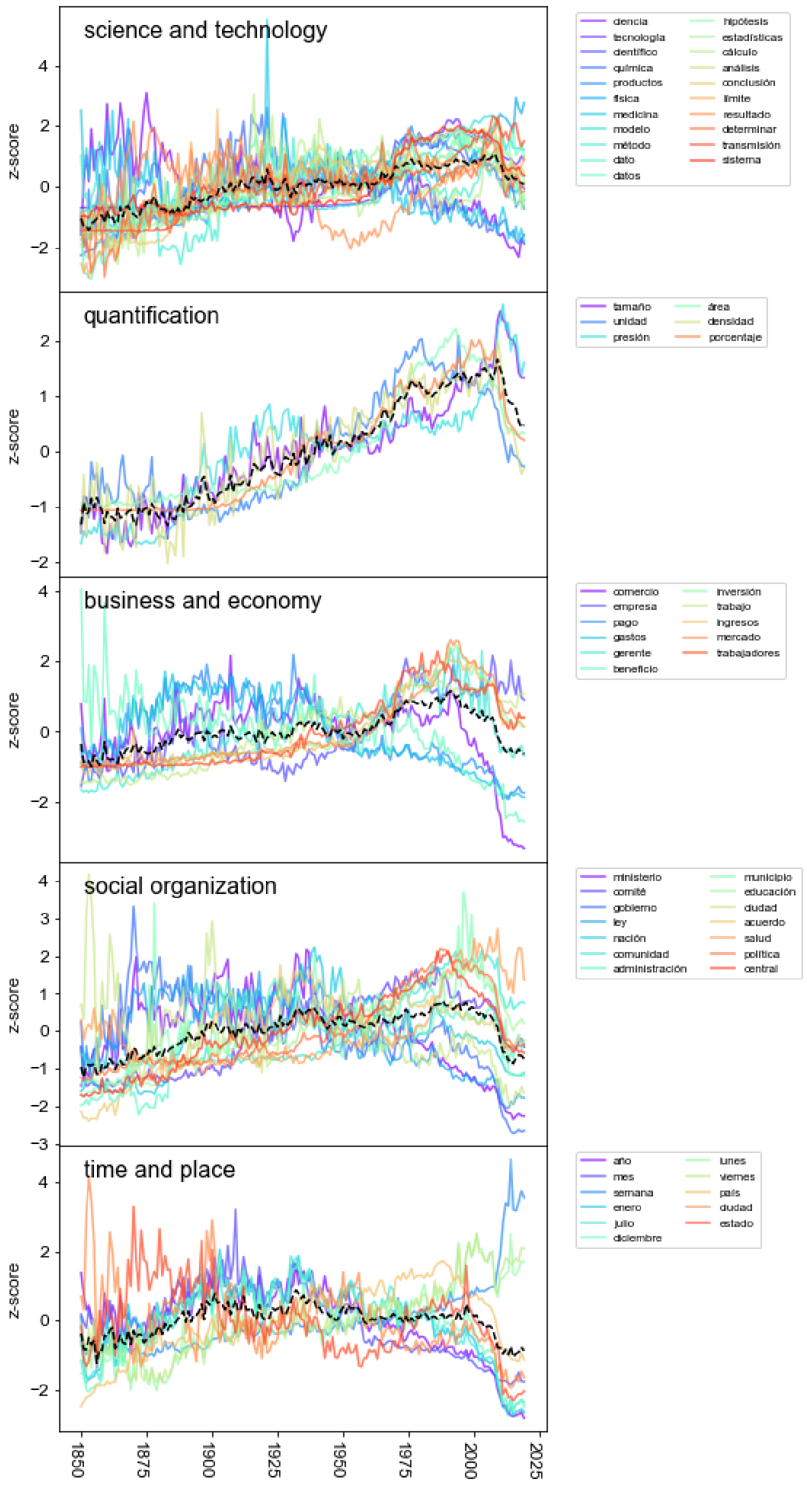

Figure S5. Time development of the frequencies of separate groups of Spanish society-related flag-words (see text above) in Google ngrams. The central black line represents the mean.

Two clusters related to cognition modes

Lastly, we explore the idea that the pe*rsonal* versus *society* groups of words may also correspond to two types of thinking that have been shown to represent fundamentally different cognitive modes of operation [5-8]: system-I ('thinking fast', loosely *intuition*) vs system-II ('thinking slow', loosely *rational*). To slightly tailor our word groups to this end, we turn the personal related cluster into an *System-I* related cluster we selected *belief* and se*nses*. To turn the society related cluster into a *System-II* related cluster we selected words related s*cience and technology*, and *quantification.*

System-I flag words thus become words related to

1) Belief
2) Senses

Resulting in: *spirit, imagine, wisdom, wise, hunch, mind, suspicion, believe, think, trust, faith, truth, true, belief, doubt, hope, fear, life, soul, heaven, eternal, mortal, holy, god, pray, mystery, sense, sensation, feel, soft, hard, cold, hot, smell, foul, taste, sweet, bitter, hear, sound, silence, loud, see, gaze, glimpse, light, dark, bright*

System-II flag words are now related to:

1) Science & Technology
2) Quantification

Resulting in: *science, technology, scientific, chemistry, chemicals, physics, medicine, uncertainty, model, methodology, method, fact, data, hypothesis, statistics, math, analysis, conclusion, replication, limit, result, determine, transmission, assuming, system, size, unit, pressure, area, density, percent*

Dynamics of those clusters are presented in Fig. 1 of the main text.

5. New York Times data

Preprocessing

We used the Archive API of The New York Times (https://developer.nytimes.com/apis), to retrieve the number of articles in which a word occurs in a specific year, for all selected flag words. To correct for the volume of articles, we scaled the time series dividing word frequencies by the frequency of an empty word ‘’. Common words like ‘the’ and ‘an’ were problematic, because they return 0’s in certain timeframes. Searches with an empty word follow the same frequency pattern as ‘the’, therefore we use ‘’ as the scale-word (Fig S6). As with the Google books data, we centered and scaled the relative frequencies to their standard deviation. The resulting z-scores were used to perform all analyses.

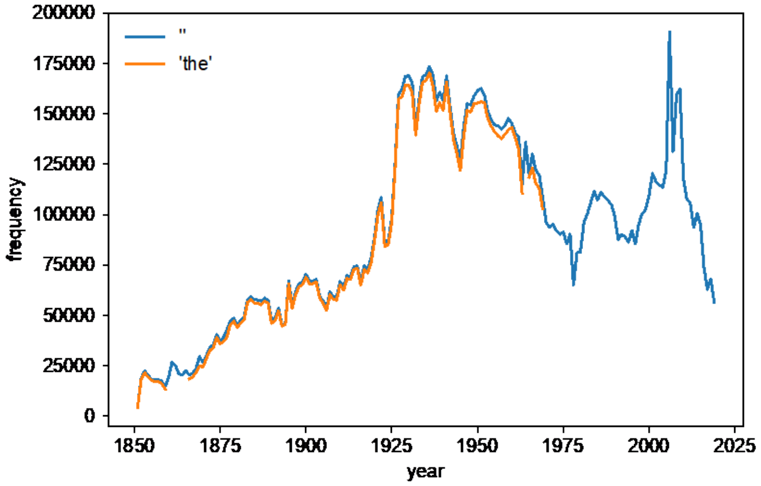

Figure S6. Yearly frequencies of words used in The New York Times for the words ‘the’ and the empty word ‘’

Personal related word categories

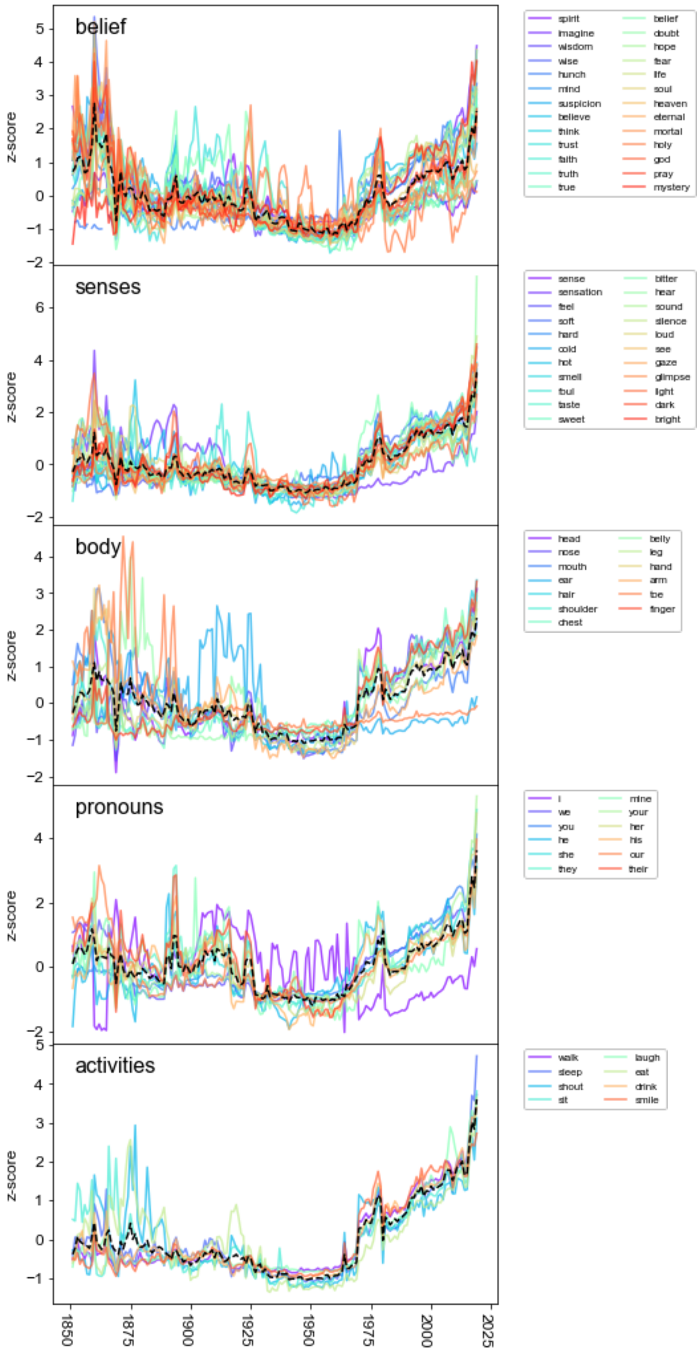

Figure S7. Trends in personal-related word categories in New York Times (compare to fig S2)

Society related word categories

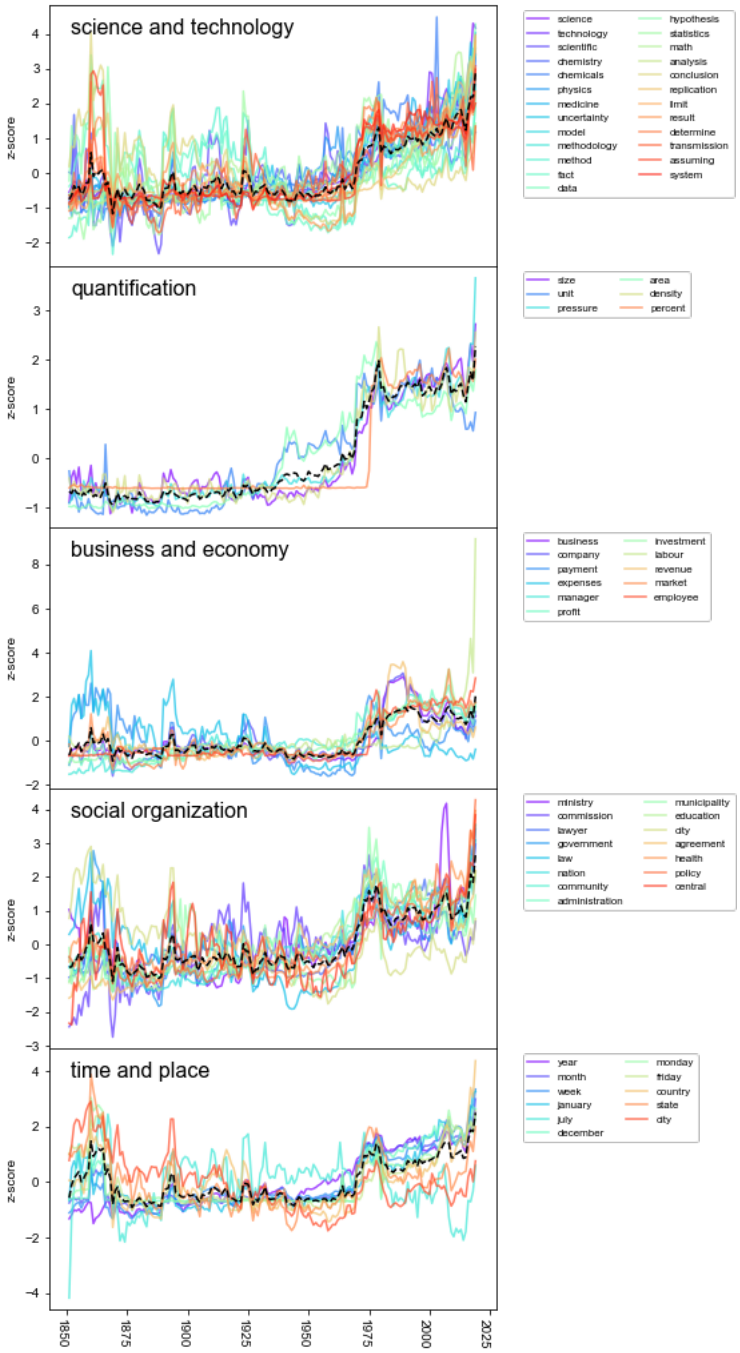

Figure S8. Trends in society-related word categories in New York Times (compare to fig S4)

6. Fiction and non-fiction

Preprocessing

The English Fiction 2019 and 2009 corpora were retrieved in the same way as the English corpus. According to Google, the Fiction corpora represent “books predominantly in the English language that a library or publisher identified as fiction”. We assume that the Fiction corpus is a subset of the English corpus. To retrieve a new non-fiction corpus (English excl. Fiction), we subtracted the raw frequencies in the English Fiction corpus from the raw frequencies in the English corpus for each word in the 5000 most frequent word list. Next, we calculated the relative frequencies, and z-scores as before.

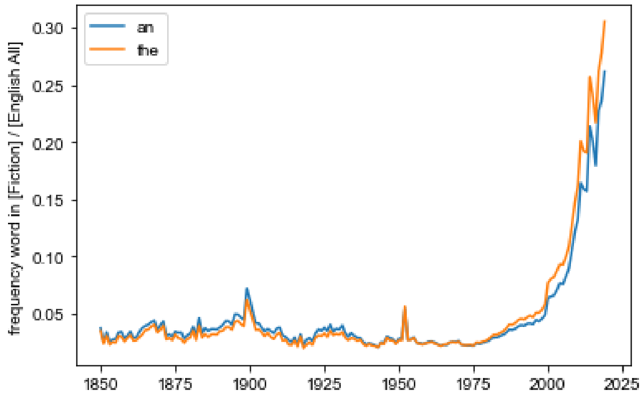

Figure S9. Proportion of Fiction in 2019 corpus for the common words ‘an’ and ‘the’.

Fiction: personal related word categories

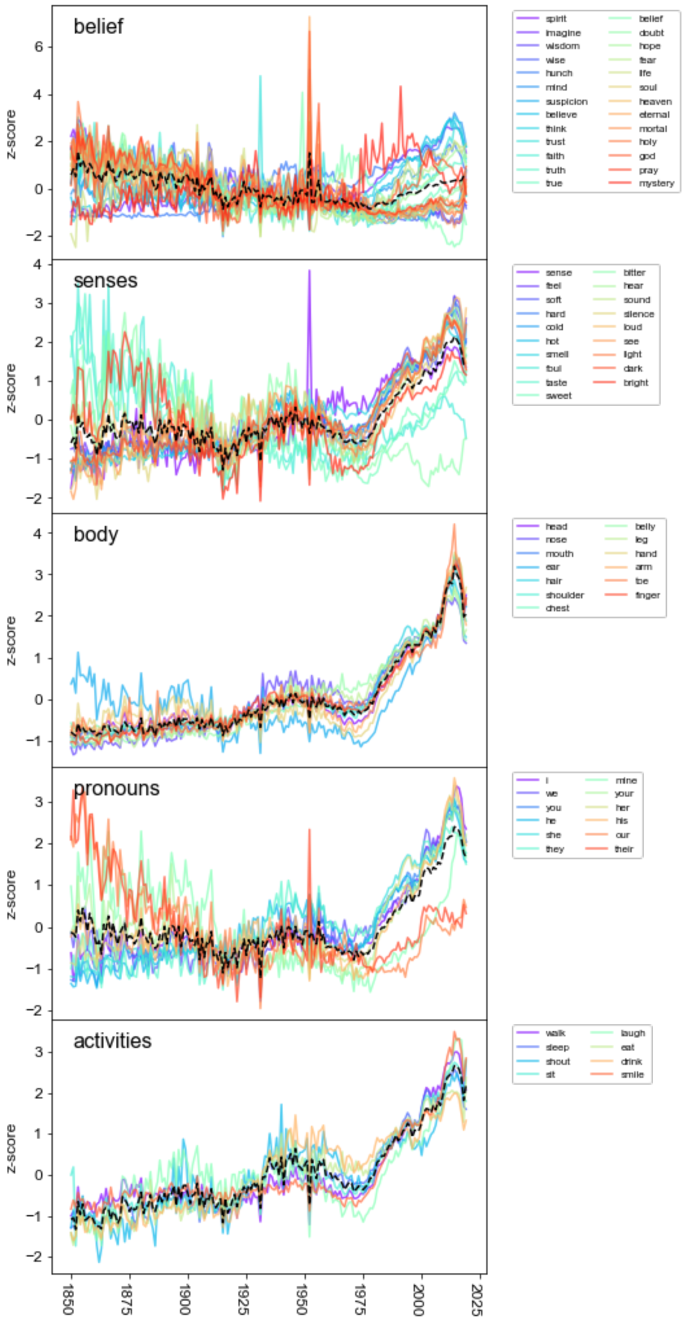

Figure S10. Trends in personal-related word categories in Fiction corpus (compare to fig S2)

Fiction: society related word categories

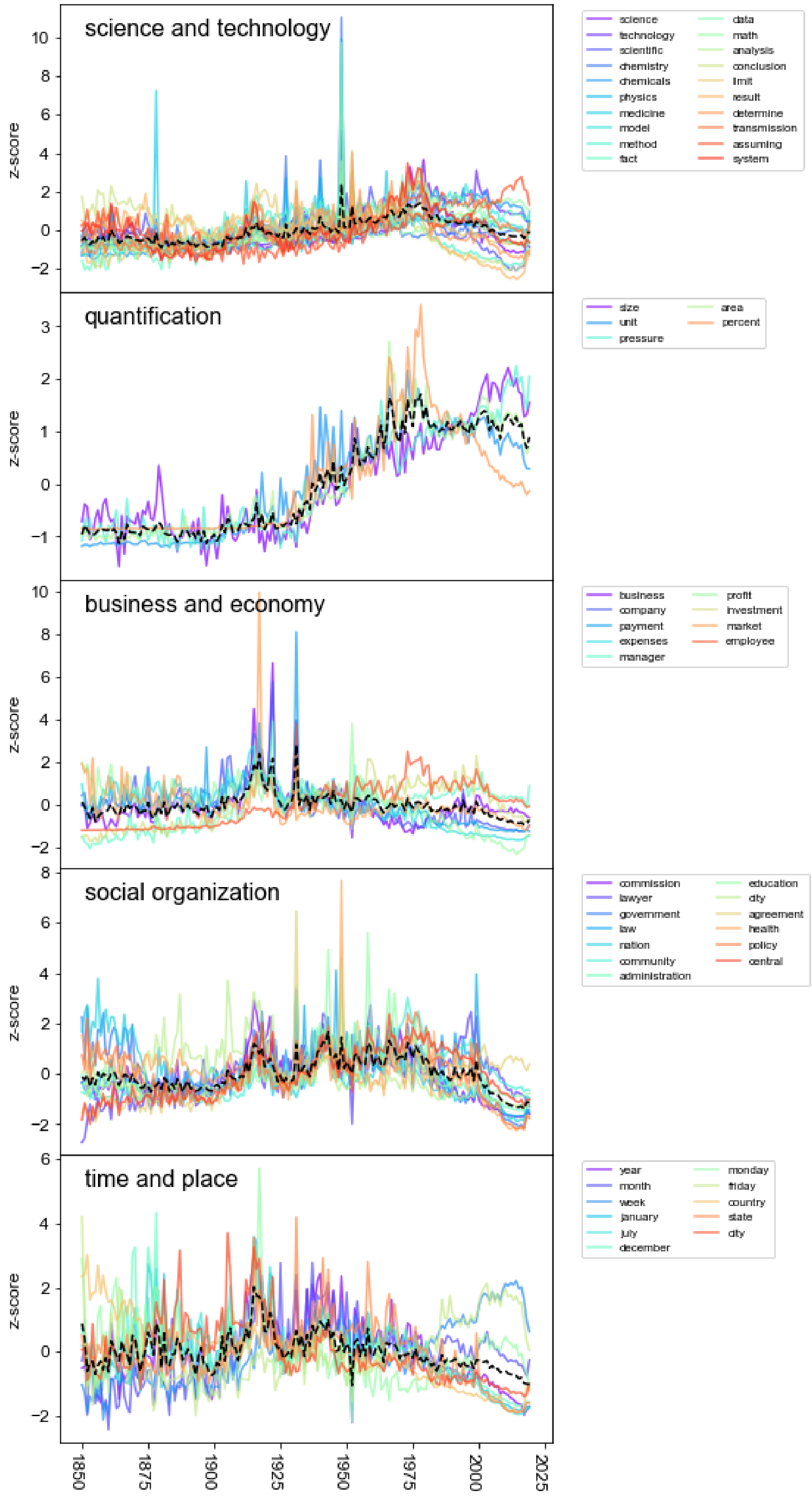

Figure S11. Trends in society-related word categories in Fiction corpus (compare to fig S4)

English excl. Fiction: personal related word categories

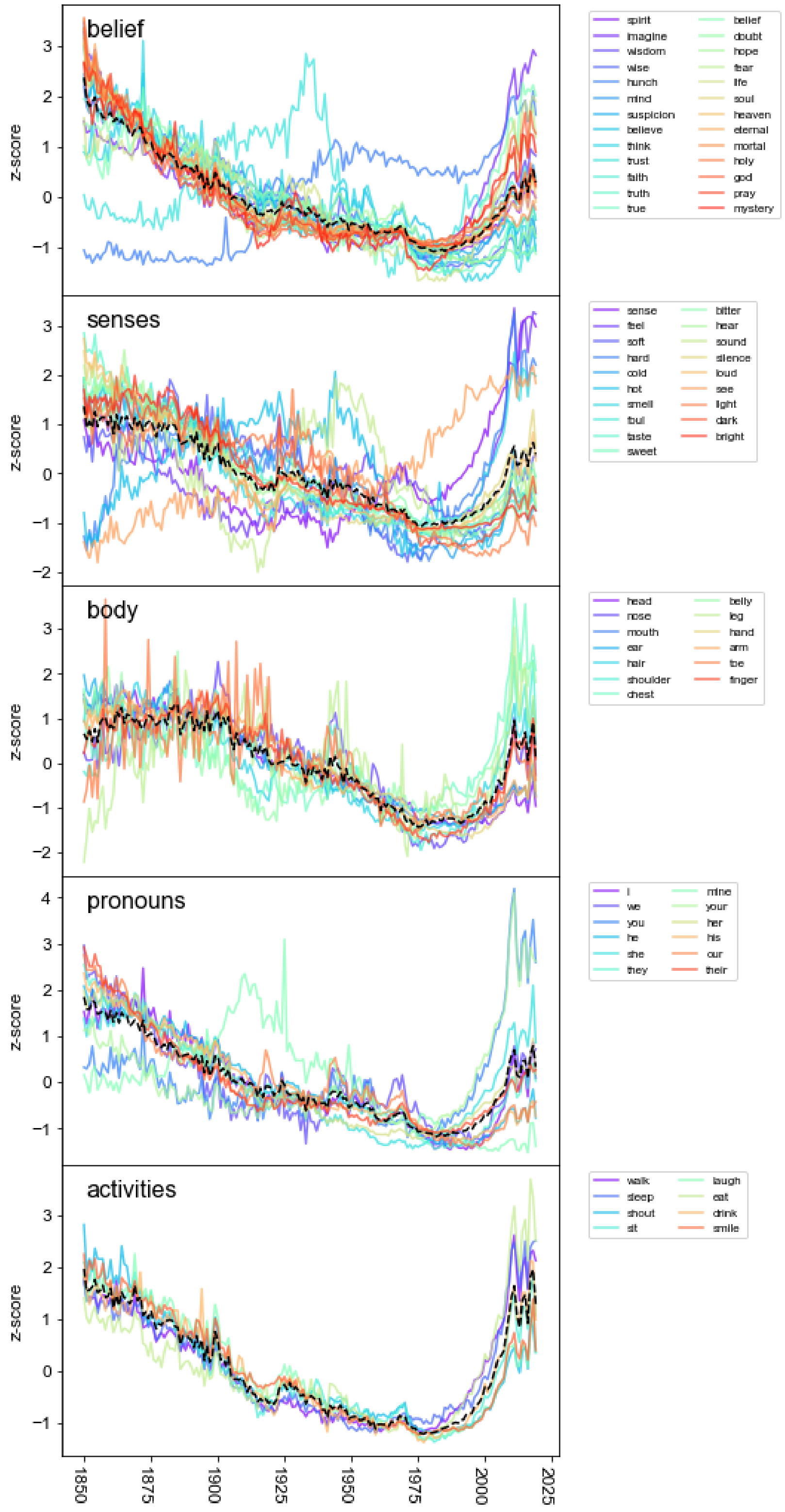

Figure S12. Trends in personal-related word categories in English corpus excluding Fiction (compare to fig S2)

English excl. Fiction: society related word categories

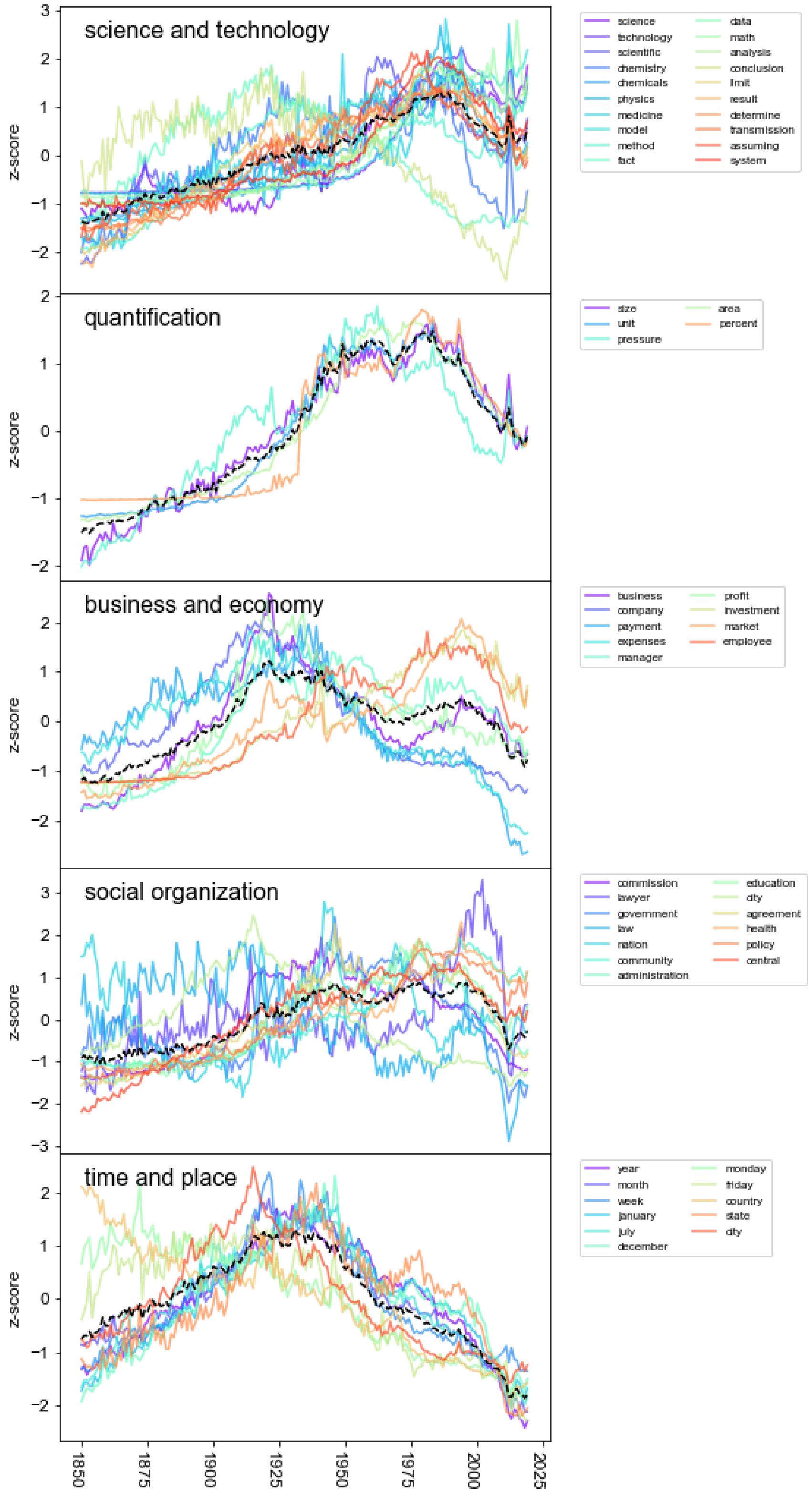

Figure S13. Trends in society-related word categories in English corpus excluding Fiction (compare to fig S4)

7. Google books 2009 data

Preprocessing

The English 2009 corpus was retrieved in the same way as the 2019, and the same preprocessing steps as described in section 1 and section 6 (for fiction and non-fiction) were followed.

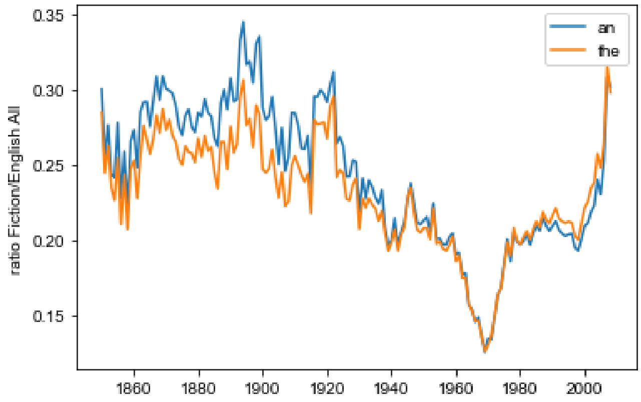

Figure S14. Proportion of Fiction in 2009 corpus

PCA, sentiment, intuition and rationality words

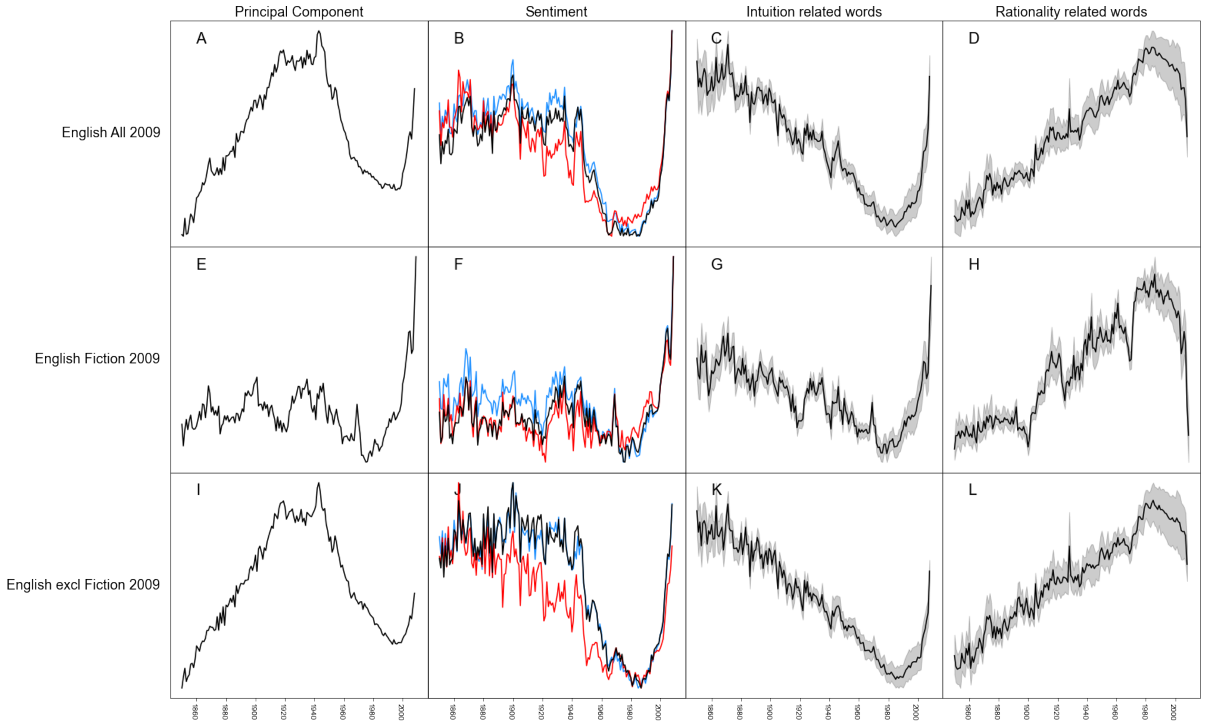

Figure S15. *Dynamics of four characteristics of English book language represented in the Google n-grams 2009 database. A, E, and I) Second principal component of change in frequency of the 5000 most used words. B, F, and J) Relative level of arousal (black), positive sentiment (blue) and negative sentiment (red). C, G, and K) Z-scores of frequencies of flag-words related to intuition, believing, spirituality, sapience: spirit, imagine, wisdom, wise, hunch, mind, suspicion, believe, think, trust, faith, truth, true, belief, doubt, hope, fear, life, soul, heaven, eternal, mortal, holy, god, pray, mystery, sense, feel, soft, hard, cold, hot, smell, foul, taste, sweet, bitter, hear, sound, silence, loud, see, light, dark, bright.. The black central line represents the mean, and the grey shaded area the 95% confidence interval of the mean. D, H, and L) Similar but for flag-words related to rationality, science, and quantification: science, technology, scientific, chemistry, chemicals, physics, medicine, model, method, fact, data, math, analysis, conclusion, limit, result, determine, transmission, assuming, system, size, unit, pressure, area, percent.*

Singular versus plural pronouns 2009 corpus

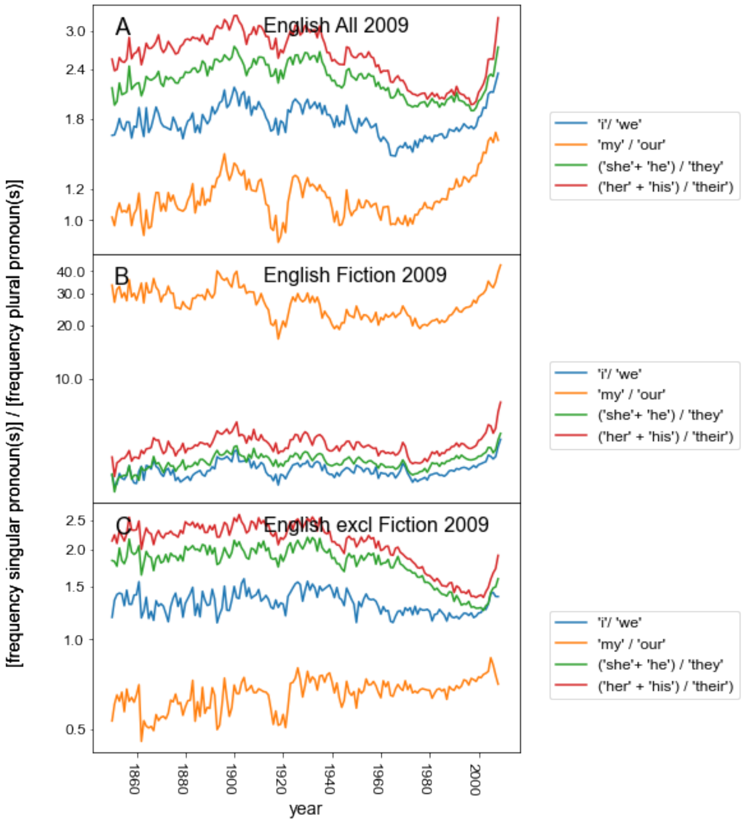

Figure S16. Ratio of the relative frequencies of singular to corresponding plural pronouns in books represented in the 2009 Google n-grams database.

Ratio between rationality and intuition words 2009 corpus

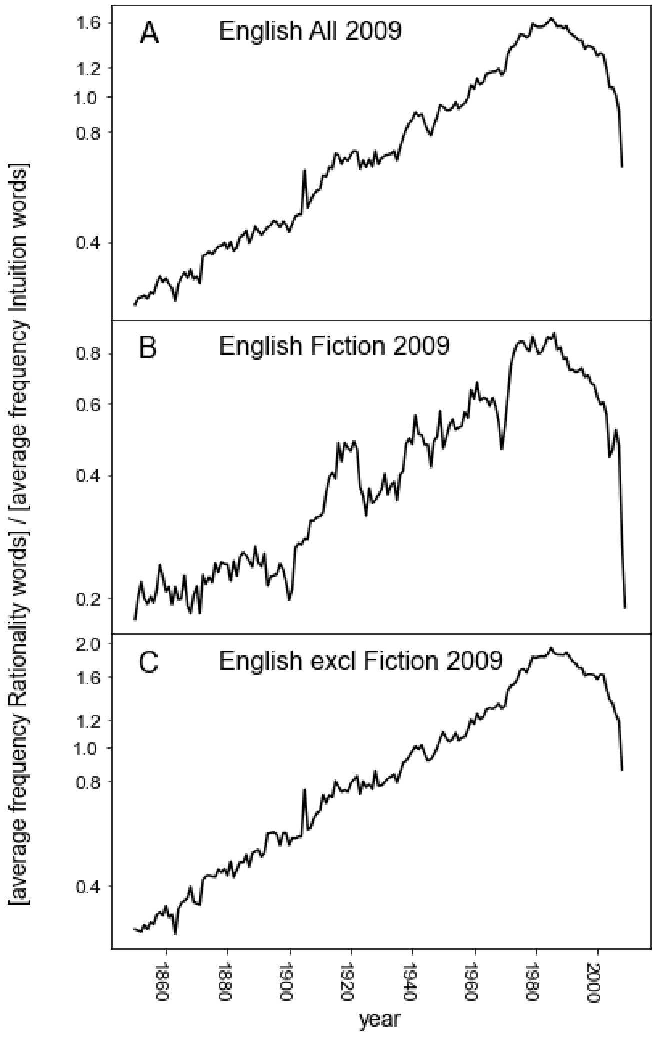

Figure S17. Ratio of intuition to rationality related words in the 2009 Google n-grams database. The graphs depict the mean ratio of the relative frequencies of the sets of rationality-related and intuition-related flag words presented in Fig. 1 (main text).

8. Google trends data

Pre-processing

For Google search trends we downloaded the frequency of each word in our 5000 most frequent words list from trends.google.com with a monthly resolution. The frequencies in the original data are already scaled such that the maximum in each time series is 100. To allow comparison to the ngrams time series, we calculated the annual means.

Null-model

To get an estimation of how word use in books reflects people's interest in terms of search behavior on the internet, we investigated whether the frequency of words tended to have the same trend in each of the two data sources. For this, we calculated the Spearman rank correlations between the annual frequency of word use in the ngrams data (e.g. 'heart'), and the annual frequency of Google searches of that same word ('heart) in the Google Trends data. To estimate how likely it is to find the overall pattern of correlations, we generated a null model in which the 5000 words in books were randomly matched to 5000 words in the google trends timeseries, with replacement. We used this null model to simulate 1000 correlation distributions. We divided the correlations into 50 bins of equal size. For each correlation bin, we calculated the mean frequency, the 5% percentile and 95% percentile. In Figure 4 of the main text we plotted the difference between the observed frequency of correlations and the mean frequency of the null-model null model.

9. Google books trends in other languages

We retrieved the google n-grams of 7 languages from the Google Books Ngram Viewer: books.google.com/ngrams (case-insensitive, and smoothing 3). We used google translate to translate the words in our subset of Intuition and Rationality word (see legends in Figures S18 and S19 for the word lists.

To retrieve relative frequencies, we chose a general word in that language as the scale word (Table S2).

Table S2. Corpora and scale words used to analyze trends in other languages

	American English	British English	German	Spanish	Italian	French	Russian
Corpus	eng_us	eng_gb	ger	spa	ita	fre	rus
Scaleword	*an*	*an*	*ein*	*la*	*un*	*un*	и (can be translated to '*and*')

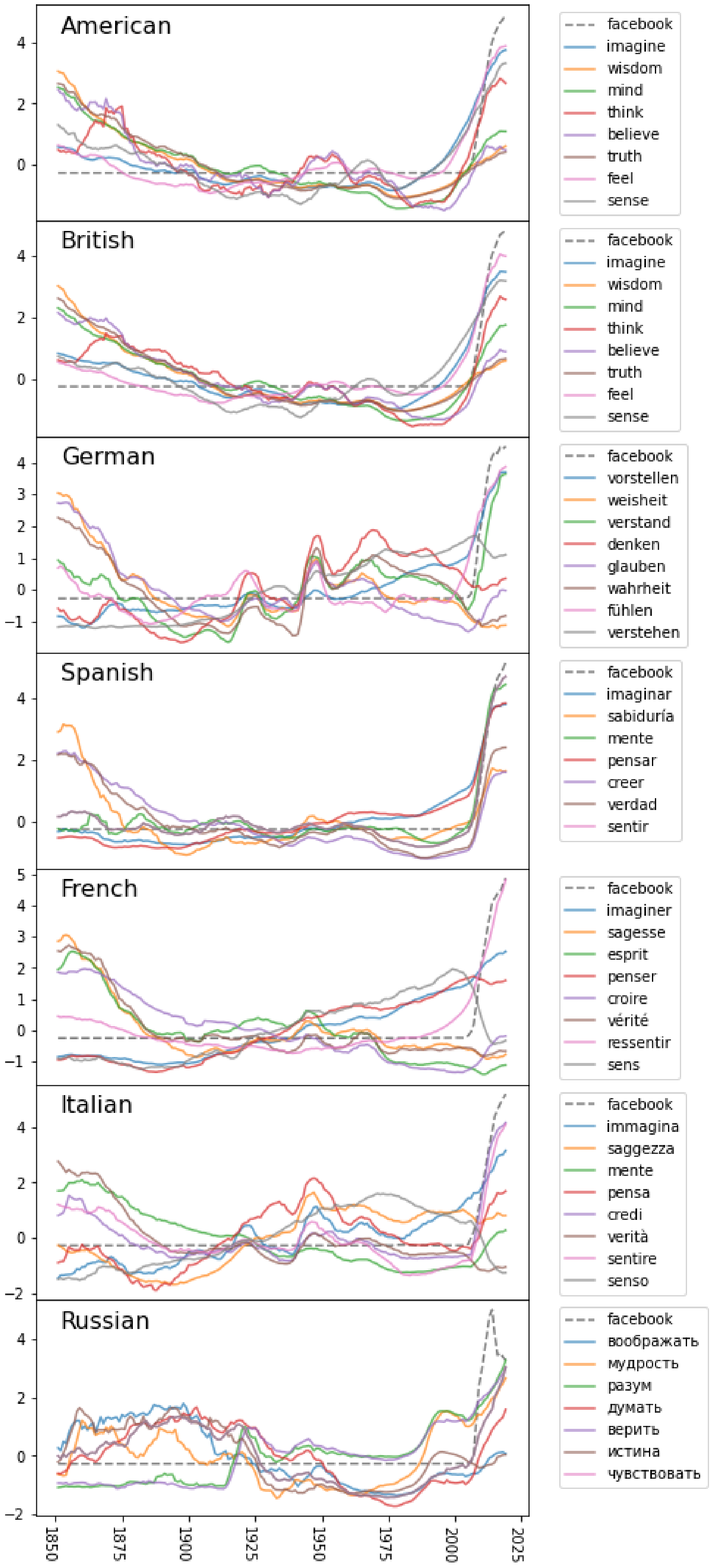

Figure S18. Z-scores of relative frequencies of a subset of Intuition words in 7 languages, and the word 'facebook'.

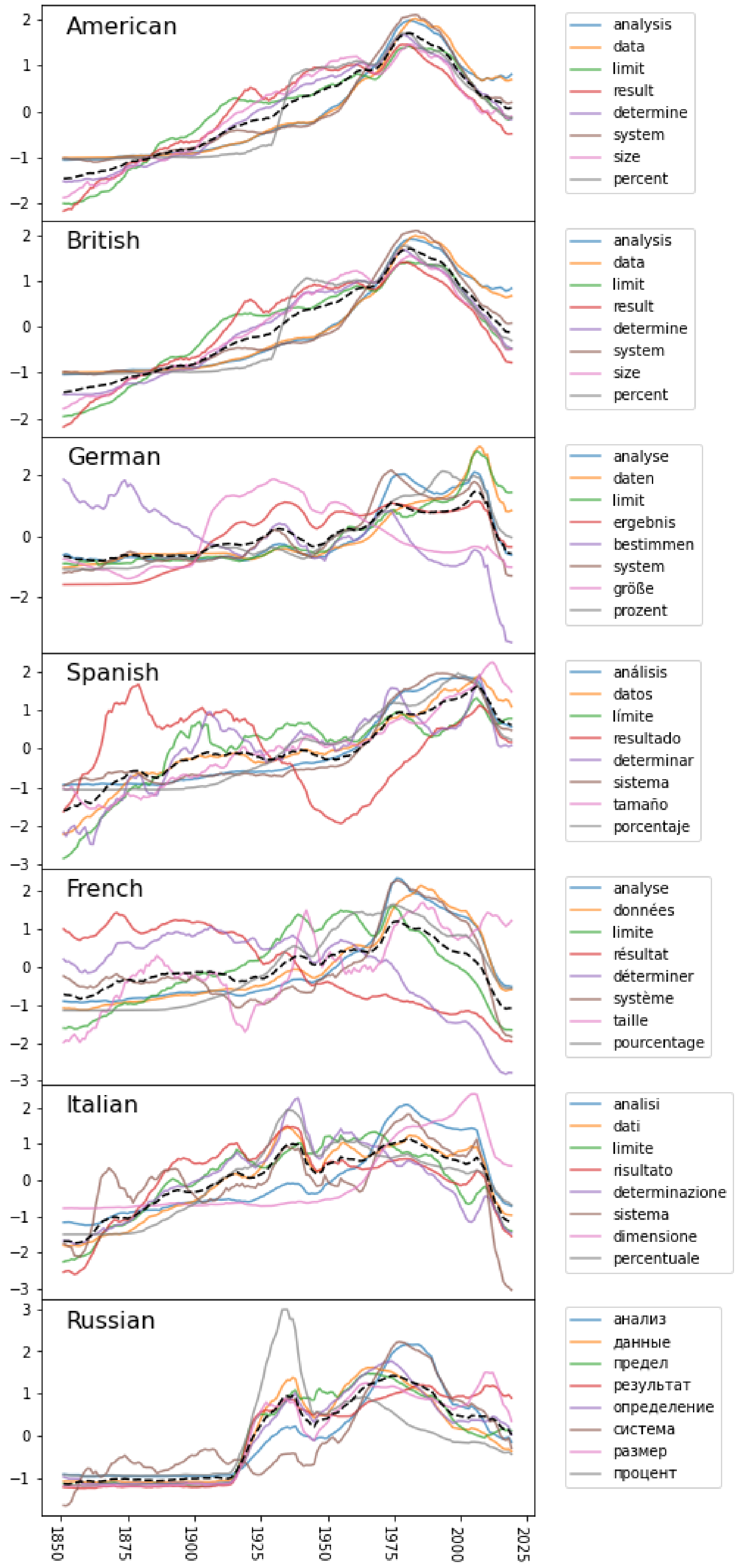

Figure S19. Z-scores of relative frequencies of a subset of Rationality words in 7 languages.

10. Top 5% words correlated to PCA, sentiment, and U-pattern

Word lists as in Table 1. Of each Google ngrams database, we listed the words that score highest vs lowest on the first and second PCA axis, the words that correlate most positively vs negatively with positive sentiment, and the words that increased most clearly after 1980 while declining between 1850 and 1980 vs words that show the opposite pattern (ranked to the absolute difference in Kendall tau in those periods). We used positive sentiment for computing the correlations, but this is closely correlated to negative sentiment and arousal.

English All

5 % words scoring highest on first PC axis (PC1):

laid, great, twelve, evidently, prove, upon, plain, mere, passing, alike, pass, very, nearly, character, sent, brought, doubt, thousand, civilized, without, same, hundred, wise, wit, intention, tide, sympathy, shore, being, useless, last, motive, habit, constantly, bore, bound, possession, cause, bark, town, hollow, executed, appearance, ground, impress, profession, ought, none, harmony, pure, opposite, altogether, worthless, hesitate, sides, unfortunate, fifty, cure, satisfied, belong, danger, drawn, against, brilliant, whole, near, nor, shade, difficulty, fatal, cast, hopeless, crown, attend, worthy, house, passage, bear, ox, kindly, seven, no, manner, every, superior, satisfaction, execution, declare, oath, acquaintance, forty, proof, succeed, proved, thrown, amusing, custom, regret, sum, twenty, crowded, quarrel, pleasant, fail, eight, beg, perfectly, poison, farther, widow, raised, cloth, usual, delicate, safely, whom, interfere, afford, fourteen, whose, true, fellow, day, occasionally, extreme, settled, carriage, sir, expedition, place, remarkable, magnificent, sheriff, give, which, maiden, valuable, disturb, chap, royal, possess, highest, account, besides, fortunate, flower, possessed, far, half, ashes, defeated, substance, guilty, eighteen, plead, delightful, fancy, peculiar, master, sons, settle, heads, dull, impression, voyage, sailing, eleven, honor, strictly, all, lie, congratulate, straw, beautifully, ours, mention, ten, scotch, ordered, applause, wish, refuse, ladies, much, found, narrow, desire, regard, thirty, pains, admitted, fall, sworn, duty, its, presume, read, merry, horse, grave, grand, sixty, have, monday, obliged, instance, afterwards, round, discovered, admit, sixteen, sickness, messenger, baggage, works, savage, bishop, mistaken, either, forth, liable, distance, evening, deed, intelligent, hardly, declared, advantage, occasion, assured, relieve, perfection, entering, and, servant, worn, till, forbid, knight, rank, witness, anxious, petty, genius, itself, sail, fond, stone, complain, gentleman

5 % words scoring lowest on first PC axis (PC1):

develop, needs, presentation, including, product, university, adult, specifically, important, replace, international, suggest, directly, discuss, location, solve, between, use, cycle, scope, include, technically, background, depending, scored, testing, routine, physical, lethal, professional, travel, primary, ability, intact, involve, log, comment, pool, involved, status, table, logical, film, lack, individual, stress, formal, alpha, initial, recognize, timer, specific, expensive, group, possibility, incoming, nursing, problem, adjust, zero, shift, biological, element, phase, today, scientist, investment, social, card, senior, leader, low, physically, investigating, heavily, difficult, camping, confusing, policy, spectacular, provide, facing, psychological, factor, winner, objective, technique, financial, activity, panel, naive, decision, split, function, g, stable, gain, documentary, project, assume, beach, helpful, definition, theater, overnight, recording, test, effective, health, contact, concentrate, photo, transmission, final, affect, sample, react, chi, b, replacement, single, typical, normally, label, community, center, also, experience, bus, campus, quality, tend, zoo, locate, x, concern, because, aggressive, setting, hamburger, ski, compete, higher, dramatic, control, listed, future, source, cigarette, clearly, plot, identification, current, based, drive, investigate, dummy, independent, working, level, student, wave, vehicle, relationship, research, treatment, frequency, development, v, study, personal, negotiate, scale, review, unique, blocking, job, backing, twin, enforcement, faced, downtown, tool, cola, presidential, visual, trading, stage, improve, grid, responsible, damage, tactical, timing, significant, breakdown, allergic, psychology, core, disagree, nearby, investigator, complex, release, seeking, achieve, drugs, spending, information, range, employee, particular, active, unfortunately,

encourage, transfer, used, structure, design, envelope, peak, internal, growth, shrimp, phoenix, parking, failure, plaza, survival, plus, package, suggesting, available, safety, tech, concentration, prime, analysis, complicated, behavior, determine, photographer, task, flow, rhythm, blocked, represent, swimming, finding, police

5 % words scoring highest on surging PC axis (PC2):
angry, look, walk, unexpected, sleep, voice, imagine, embarrassed, tortured, heal, struggling, knowing, potion, ambush, incredible, looking, greedy, terrified, looks, how, torture, learn, anger, invisible, mother, comfortable, drunk, fade, like, brutal, harsh, yourself, pain, sofa, could, dream, distracted, crying, what, thanks, her, eat, walking, shower, helmet, warn, suspected, sense, luckily, smell, monster, forgiveness, leap, awkward, breathing, couch, breath, wrong, champagne, ride, asleep, bastard, listen, hate, confident, emotion, chose, sitting, your, compassion, breakfast, beat, literally, expression, laughing, smiling, drink, tom, sat, she, felt, hang, hide, call, idiot, sit, joke, waking, belly, watch, wait, gently, led, smile, spit, suspicious, breathe, hair, kissing, suck, inspired, lose, gig, alive, whore, drove, remind, follow, unlock, enjoying, stairs, knew, hers, kiss, convince, smack, told, planet, anxiety, wakes, hint, even, vomit, flirting, often, liar, else, sky, intense, hidden, did, stupid, jenny, saying, ugly, bracelet, treat, hell, surprised, whew, favorite, instead, eyes, barking, nap, sneaking, door, herself, yell, sorry, pointing, uncomfortable, breasts, dragging, accent, hah, scream, scent, mock, stalking, tore, pillow, shake, whispering, rang, magician, becoming, remember, laughter, oh, hanging, chat, lust, watching, nowhere, spy, cheek, hunger, grew, tell, forever, limo, bleeding, polite, swim, toss, knocking, spice, silence, blew, demon, watched, necklace, shy, knee, threatening, turned, witch, laugh, lied, behind, feel, mummy, face, tasted, josh, you, sure, catching, worst, warrior, eve, off, lazy, wore, blame, dragged, steal, beating, aha, easy, surprise, turns, silly, window, harm, inviting, me, clock, powerful, woman, neck, shelly, hungry, ass, shook, universe, stab, shaking, chest, shot, heather, meant, memory, bloody, stare, gossip, why, whoever, stuffed

5 % words scoring lowest on surging PC axis (PC2):
secretary, state, report, year, sec, council, order, authorized, district, west, eastern, behalf, northern, president, office, statement, under, january, vice, attorney, east, committee, resident, october, south, reference, officer, branch, annual, interest, prepared, following, commonwealth, august, counsel, exclusive, further, board, april, collected, november, february, july, jersey, september, jurisdiction, general, contract, permanent, remaining, atlantic, salary, greater, less, applied, construction, assistant, supply, other, separate, december, june, north, commissioner, fund, public, meeting, reasonable, member, uniform, purchase, lease, transferred, operation, inspection, soil, special, southern, month, elsewhere, permit, various, depth, paper, congress, number, price, copy, effect, water, experiment, discharge, provided, united, deputy, goods, march, gross, record, chairman, except, extension, position, deposit, college, advise, approve, during, agree, settlement, aid, authority, necessary, circuit, testimony, col, joint, certificate, dam, corporation, actual, division, weight, copper, demand, minister, department, stud, complete, previous, session, in, trade, importance, bull, station, gas, crane, steel, lower, per, title, pacific, reserve, chemistry, lake, below, payment, total, tube, plant, dealt, amount, each, acid, receipt, these, average, expenses, government, strike, supreme, capital, hopper, absence, tobacco, regular, nickel, association, fairly, original, assigned, established, form, commission, accepted, article, warren, action, western, cost, shipping, mutual, increase, pump, representative, electric, temporary, farm, dealer, clay, covering, obligation, customs, weed, recently, pine, accident, act, license, insurance, contain, investigation, san, institution, conclusion, union, mines, senator, sale, sand, loan, bureau, engineer, charging, shown, gravity, creek, shipment, appeal, alcoholic, ridge, convention, valley, memorial, closely, miller, sub, minor, dean, submit, service, announcement, principal, feeding, shallow, highway, labor, feed, prosecution, county, testify, progress, hospital, material, net, complaint, passenger, continued, unless

5 % words correlating most positively to sentiment:
dressed, nights, beating, mad, forget, perfume, wore, delicious, crowd, dinner, took, sister, whispering, saw, hung, next, shut, bad, together, suddenly, slept, beside, thought, away, stood, another, awake, spoke, alive, drank, me, down, broke, dark, blame, inviting, whisper, drown, too, polite, moment, dragged, life, hang, quietly, forgot, glow, silence, footsteps, surprised, forehead, lose, laugh, lazy,

lonely, shake, again, sit, stir, i, turning, body, silent, fool, tasted, steal, head, shout, forgetting, my, seeing, song, instead, sky, babe, wonder, loud, drink, herself, liar, tea, warm, surprise, bed, into, uncle, touched, mouth, cursed, neck, tore, then, cheek, gorgeous, gone, still, went, asleep, afraid, stole, oh, find, stairs, invite, threw, hint, breakfast, not, hurry, lived, gossip, left, night, naughty, drew, laughing, hanging, begging, tell, breathe, knife, gown, aunt, eyes, pillow, always, calling, mother, pause, worse, suspicious, sink, forever, shadow, conversation, here, let, her, beat, whoever, forgive, mystery, waiter, horrible, sitting, pirate, frightened, thing, naked, annoying, grew, dress, feeling, lovely, swallow, knowing, drove, arm, pretty, rub, horror, gently, quiet, furious, wasting, waking, forward, take, what, boots, enjoying, eve, come, rob, desperate, hand, taking, walk, struggling, ringing, watch, sat, marry, liking, marrying, ugly, distracted, eats, robe, smiling, engagement, when, disappoint, rushing, teach, greedy, love, dawn, tear, stray, remember, pistol, sleep, worst, mate, dead, donkey, imagine, shot, jewel, chill, laughter, darkness, soft, ridiculous, knew, go, knee, thanks, voice, riding, underneath, crush, eaten, drunk, idea, if, barking, purse, came, blew, thirsty, sleepy, told, invisible, nowhere, monster, singing, murderer, warrior, ahem, happy, saying, lily, lip, hear, gather, pudding, enough, couch

5 % words correlating most negatively to sentiment:
deputy, separate, annual, surface, applied, report, joint, contain, sub, marine, effect, determined, counsel, established, foreign, reasonable, congress, qualified, gross, number, direct, violation, assigned, tables, increase, request, section, savings, remaining, temperature, library, permit, construction, funds, reference, chemistry, transportation, manual, provided, volume, capital, chemical, assist, public, member, retarded, demonstration, affected, department, rate, office, p, depth, statement, relative, establish, lower, cost, laboratory, title, total, state, draft, chairman, loan, application, council, government, base, minor, liquid, executive, agreement, editor, identical, commercial, fuel, engineering, congressman, administration, civil, employment, proposal, dollar, assumed, l, apply, gas, terminal, result, representative, medical, indicate, article, register, hearing, assistance, commission, electrical, tax, conference, scheme, maintenance, previously, western, technical, probation, director, special, shallow, trade, investigation, federal, schedule, approval, vacuum, considered, fund, benefit, tested, division, record, price, telephone, equipment, maximum, pa, limit, limited, pressure, shown, demand, require, assuming, minister, automatic, additional, addition, maintain, secretary, solution, period, scientific, generator, service, president, notify, authority, united, burden, union, further, size, finance, official, industry, national, central, similar, operating, constant, contract, meter, prior, determine, registration, minimum, employer, private, basis, cable, experiment, transcript, operation, capacity, nickel, officer, handling, strain, associate, are, san, extension, for, storage, material, col, item, various, income, sec, transfer, attorney, inspection, calendar, column, registered, sales, obligation, education, personnel, operate, rice, procedure, recent, concerned, million, plant, metal, soil, recently, fishing, october, included, area, september, graduate, insurance, anniversary, guarantee, field, fee, chemicals, classified, atlantic, average, circuit, studied, method, program, producer, repair, aid, zone, fish, accurate, credit, budget, net, association, local, assignment, physics, during, award, alcoholic, retirement, unit, isolated, ridge

5 % words rising after 1980 and declining before that year:
perfect, understood, throw, them, embrace, sight, comfort, nothing, rushing, place, trusting, awful, beautiful, ever, hearts, never, awake, throwing, when, sweet, promise, fallen, threw, cheer, brother, so, spirit, breathe, every, owe, believing, thankful, footsteps, him, rest, stranger, gorgeous, seeing, supposed, ashes, surprised, joy, cheering, disappoint, stood, thrown, dare, who, shine, appetite, desire, escape, disappointment, honest, mind, hear, eye, jealous, into, together, excuse, mistaken, perfectly, ashamed, horrible, lay, my, accidentally, forgotten, her, slept, patience, then, calm, supper, forgot, cheek, tongue, us, admit, surprise, beloved, kneel, ruin, happily, thing, soon, moment, cry, truly, myself, saved, instead, ruined, smile, glory, walk, forget, bless, lost, selfish, gentle, strange, whisper, saw, dead, mercy, wander, framed, breeze, excitement, me, shadow, deserved, stole, body, darkness, humanity, pleasure, true, forgetting, suffering, journey, mighty, silent, touching, forehead, thirsty, loud, seat, flesh, yourselves, beneath, they, but, heart, wanting, he, eyes, glorious, taught, dress, calling, father, breath, his, wandering, disappointed, soul, faithful, prayer, crowded, let, quiet, silence, endless, blessed, evening, fell, happiest, shut, took, truth, safely, broke, apology, drew, draw, herself,

delight, sober, heaven, ambition, lighten, brave, distant, stairs, death, heavens, gave, shake, feeling, forgive, gather, yet, sad, evil, dragged, deadly, night, asleep, wisdom, wonder, alone, suspicion, honesty, steal, surrounded, shame, hung, alive, imagine, voice, punish, shed, once, praying, sister, pretend, terrible, shout, sake, pray, inviting, blessing, shining, leaving, gone, happiness, mysterious, fit, knowing, miserable, quietly, sorrow, feared, their, couch, suddenly, eager, away, beast, rude, foolish, tasted, precious, sing, babe, courage, bread, hang, dreadful, beyond, swore, adore, kindness, perfection, obey, themselves, head

5 % words rising before 1980 and declining after 1980:
area, program, indicate, available, development, basis, determine, initial, technical, million, addition, final, range, replacement, personnel, control, unit, involved, percent, eliminate, limited, rate, concentration, increase, result, test, staff, included, tested, transfer, maximum, zone, plus, sample, recent, congressman, level, funds, data, responsible, basic, laboratory, equipment, budget, procedure, breakdown, effective, activity, tape, review, nuclear, factor, federal, base, prior, testing, chemical, isolated, system, minor, designed, automatic, analysis, decision, marine, significant, chemicals, storage, growth, limit, separate, peak, manual, represent, sophisticated, minimum, occur, approximately, potential, administration, failure, higher, savings, maintenance, b, size, similar, current, flow, engineering, direct, primary, chairman, directly, policy, study, tables, temperature, production, identical, billion, vacuum, radiation, recording, package, national, objective, standard, training, concerned, scale, additional, effort, worker, reliable, fuel, central, representative, capacity, transportation, conference, technique, pattern, frequency, schedule, research, medium, investigator, deputy, low, handling, income, phase, assuming, transmission, hearing, problem, responsibility, annual, used, clearance, division, terminal, operating, transport, total, computer, director, physics, type, group, identification, center, department, typical, employment, gross, construction, damage, provide, volume, sub, listed, product, tax, section, proposal, electrical, retarded, affected, concern, prime, structure, approval, record, vehicle, permit, delayed, item, nursing, response, industry, dollar, aircraft, effect, sales, cycle, meter, operate, field, series, individual, determined, library, agreement, nickel, lower, normally, recovery, accurate, alternative, producer, defense, request, complete, specific, employee, surface, element, unfair, operation, major, tech, statement, constant, exposure, high, rice, comment, auto, executive, population, local, develop, load, cost, suggest, report, studied, loan, replace, education, during, p, classified, overtime, maintain, status, supervisor, relative, special, draft, material, stereo, demonstration, office, investigation, western, column, technology

Spanish

5 % words scoring highest on first PC axis (PC1):
virtud, cuyo, causa, cuya, por, cuyos, consiguiente, hechas, hacerse, formado, haberse, halla, cualquiera, dará, misma, cuyas, efecto, recibido, formar, cuanto, conveniente, decirse, mismas, merece, siendo, seguida, leyes, iguales, inútil, conservar, proceder, empeño, impedir, adquirido, partes, alguno, dar, contra, estando, ocupar, quedando, el, teniendo, objeto, al, órdenes, basta, podido, nombre, ocho, presentarse, han, seis, notable, verdadera, servido, separado, particulares, preciso, vista, verdaderamente, inmensa, dé, harán, dueño, aviso, esta, obligado, cuantos, sabido, dando, verdadero, todas, hayan, palacio, honor, cuatro, primero, tomado, graves, hecha, guarda, haya, pues, cual, circunstancias, mismo, ellas, servir, serlo, forman, quiera, notables, formando, fortuna, inmediatamente, llevando, mismos, tesoro, tercero, defectos, último, vienen, causas, formada, entera, haber, ramos, regla, nombrado, justicia, paga, doce, frutos, provincias, autoridad, ministros, dado, satisfecho, conforme, satisfacer, habido, sean, inglaterra, célebre, puntos, reconocido, toda, sea, testigos, condenado, pueda, noticia, dice, dolores, jueces, ni, seno, justa, levantar, ha, declaró, inteligencia, ventajas, cien, las, dejando, talento, semejante, adquirir, quede, fin, cualidades, deben, noble, oficio, considerable, tres, sucesos, conviene, puesto, cuales, deber, necesario, negocios, tenga, propuesto, útiles, creyó, fácilmente, presentó, pedir, medio, bajo, arreglo, veinte, conserva, hará, constantemente, pudieran, ejército, cuarta, observaciones, este, piden, grave, remedio, cometido, escrito, hubiese, llamados, tengan, hagan, uu, necesarias, favor, vencer, motivo, dos, tribunales, antiguo, alguna, cumplido, aquellas, ponga, circunstancia, todos, entregar, fuese, verá, sirven, antiguas, facilidad, necesaria, haga, completa, dueños, marina, cuantas, soldado, sentimiento, aun, semejantes, pertenecen, disponer, será, enemigo, costumbres, esperanzas, gravedad, ocupado, armas,

contienen, contiene, suceso, mando, fundamento, toca, grados, manifestó, faltan, diez, hierro, corre, falta, culto, marqués, facultades, igualmente, doña

5 % words scoring lowest on first PC axis (PC1):
surgió, profesionales, inicial, realiza, función, periodista, tipos, distintos, realizan, experiencias, mediante, grupo, crear, fracaso, imprescindible, aparentemente, realidad, capacidad, selección, concreto, profesional, cambio, comprensión, similares, visión, prácticamente, preocupación, eliminar, sentido, periodistas, adaptación, complejos, versión, eliminación, adecuado, iniciar, trabaja, actúan, basa, relación, amplio, mantenido, interpretar, actúa, mantiene, juega, equilibrio, múltiples, generación, complejo, significado, directo, decisión, tipo, realizar, factores, referencias, especialista, adecuada, futuro, encontramos, relacionadas, utilizando, bienestar, componentes, investigación, aparición, noción, posición, utilizado, expertos, colectivo, actuar, elaboración, relacionado, funcional, posibilidad, definición, utilizados, desarrolla, tarea, referencia, desarrollado, comenta, caracteriza, perfil, posiblemente, experiencia, modalidad, fase, reflejo, amplia, trabajo, relacionados, retorno, investigadores, valores, positivos, avance, negativo, colectiva, radio, fases, lógica, permiten, tema, cierre, trayectoria, ocurre, exigencia, transformación, basado, científicos, distintas, mínimo, formas, visitantes, teoría, características, compleja, hoteles, específica, entidades, miles, coinciden, montaje, mantienen, convivencia, emergencia, incluyendo, protección, categorías, colectivos, fundamental, control, positivo, mayoría, azar, equipos, ciclo, través, clásico, basada, concentración, implica, norma, elemento, episodio, desaparición, disponibles, entidad, extrema, europeo, mecanismo, problemas, desarrollar, explica, identificación, funcionamiento, intercambio, docentes, comienzo, describe, actividades, definido, creó, prefiere, integral, temas, individual, afrontar, factor, introducción, regulación, caída, tareas, ambiente, oportunidades, actitudes, concretos, meta, trabajan, vehículos, críticas, estudiantes, parcial, característica, difusión, bloque, relato, infantil, similar, destaca, centro, técnicas, totalmente, final, permite, orígenes, obtención, conjunto, expresión, situación, insiste, realizando, mantenimiento, plantea, juvenil, comienzos, contactos, requiere, prevé, familiares, personales, elaborar, educación, podrían, ritmo, contenido, núcleo, especialistas, complejidad, posibles, pese, estadio, comentarios, etapas, asumir, tradición, jean, formación, realizará, elaborado, realizadas, fundamentales, podríamos, editorial, consumidor, intervenciones, requieren, porcentaje, ruptura, símbolos, creación, new, actividad, debido, deberían, aspectos, concreta

5 % words scoring highest on surging PC axis (PC2):
hablando, acabar, buscar, dejaba, mano, esperar, tocar, respondió, cuánto, pensamientos, era, acabado, quién, pasaba, cuando, parar, salir, daba, silla, perder, añadió, comer, subió, dejó, estaban, cayó, repente, volver, vergüenza, perdido, acabó, permanecer, paso, beber, pasos, pareció, secretos, bajar, cabeza, voz, ser, sacó, ganar, rodillas, ruido, unas, ver, madre, llevado, continuó, vestido, llamas, hermana, dejar, caer, volvió, duro, deseaba, conocido, tener, añadir, mira, pero, placer, subir, dijo, puerta, dejado, qué, aunque, miradas, noche, solas, acababa, oscuridad, lo, mirar, cuál, amigos, manos, ir, padres, puso, golpe, sentado, tu, regalo, olvidado, pesar, llevaba, hablamos, eran, quedaba, mucho, hablar, fuera, gracias, tus, ira, nunca, darle, llamó, dedo, quedó, dolor, abrir, descubrir, brazo, esperaba, reconoció, cuántos, rostro, abandonar, debajo, señal, acercarse, estar, aliento, descubrió, idea, brazos, aquello, boca, empezado, pasando, ella, toque, esperando, estaba, cerrar, segura, detener, nada, suele, vaso, consiguió, parecido, rabia, nadie, romper, olor, echó, quisiera, malo, él, palabras, mentira, perdió, unos, mostró, verla, juntos, pasó, cuello, conmigo, esposa, empezaron, llamaba, gritos, ah, quedaban, leve, mi, hábitos, delante, ya, veo, puedo, metido, preguntar, instante, tampoco, sentarse, has, todo, entender, vuelto, iba, tiendas, corriendo, ves, levantó, peligroso, sí, loco, llevar, mis, hacerle, vueltas, locura, cuándo, mejor, promesa, ojos, está, llave, oyó, matar, personas, entrada, soy, segundos, me, vamos, decir, persona, cerrada, tranquilo, secreto, dices, deja, aseguró, mostrado, tú, nos, tienda, usar, detuvo, abrió, suelen, llamada, feliz, pierna, golpes, haces, llorar, ofreció, padre, loca, poco, saber, iban, te, necesitaba, le, vuelta, cosas, quiero, salió, trato, vaya, verdad, perdida, siguió, bajó, fuerte, culpa, amiga, disfrutar, oh

5 % words scoring lowest on surging PC axis (PC2):
corresponden, del, diciembre, carácter, capital, julio, correspondientes, josé, extranjeros, enero, comercio, actual, importancia, refiere, legales, marzo, corresponde, abril, octubre, noviembre,

respectivamente, mandatario, manuel, firma, corrientes, condiciones, febrero, expresa, fecha, oficial, firmas, comunicaciones, publicado, mayo, director, ejemplares, junio, agosto, angel, establecido, figuran, anuales, pacífico, representantes, exclusivamente, directores, norte, modificaciones, propietarios, progreso, extranjero, éstas, actualmente, general, chile, extranjeras, impone, exteriores, constituyen, presidencia, siguientes, gobiernos, emilio, vicente, funcionarios, agricultura, república, funcionario, valle, asamblea, garcía, tarifas, nacionalidad, rafael, toneladas, perú, ésta, agrega, secretaría, relativa, crédito, miguel, industrias, éstos, carece, muñoz, industria, estima, nacionales, definitivo, paraguay, eduardo, vicepresidente, informe, militar, mandato, alvarez, demuestra, ciento, argentino, presidente, informes, departamento, municipal, artículo, uruguay, soberanía, garantía, aires, nicolás, torres, impuestos, mariano, especial, talleres, joaquín, declaraciones, cultivo, correspondiente, referente, labores, aumento, especiales, obra, diarios, éste, alcanza, luis, santiago, guillermo, localidad, corporación, fondos, deberán, fines, concepto, adolfo, delegados, americano, ríos, campaña, exige, autoridades, exportación, chileno, lima, legal, criterio, comerciantes, independencia, argentinos, originales, colonia, banco, tránsito, intereses, mencionado, nación, cuadros, anual, dr, extraordinaria, geografía, obras, ortiz, americana, antecedentes, félix, impuesto, solicitar, agregar, rosario, deberá, buenos, escuela, delegado, representante, vigente, ejemplar, cifra, domicilio, américa, martínez, resolución, sometido, herrera, menores, reserva, peña, rodríguez, americanos, vigor, nacional, moreno, dispone, intervenir, establece, actuales, enseñanza, efectuar, tierras, industriales, cuba, comisión, bolivia, presentada, legislación, curso, agustín, definitivamente, trabajos, extensión, renuncia, extraordinario, operaciones, exigencias, consideración, ramírez, brasil, división, garantías, rivera, registro, antonio, silva, defensa, molina, manifestaciones, gómez, argentina, ingeniero, numerosos, gutiérrez, ejecutivo, aprobación, convenio, restantes, gonzález, dificultades, obreros, fiscales, núñez, proyecto, presente, asuntos, hectáreas, explotación, comisiones, domingo, pérez

5 % words correlating most positively to sentiment:
mejor, ojos, humo, tristeza, rostro, vi, silencio, mirar, llorar, habló, risa, cansancio, unos, manos, rabia, sienta, miraba, curiosidad, prisa, loca, hablaba, suave, voz, mirando, metido, dormido, regalo, tu, desnudo, vestido, siguió, tú, iba, demasiado, noches, muerta, nunca, roto, olvidado, tus, duro, eres, rato, aire, ves, pero, llegaba, soy, pequeño, gritos, apenas, verla, haces, te, bueno, echó, boca, has, detuvo, sentir, desconocido, conmigo, unas, noche, mí, vueltas, quiero, sorpresa, alegría, rodillas, ti, labios, todavía, pensaba, amiga, brazos, puerta, ventanas, habilidad, pensado, hombros, beber, ir, oreja, sentarse, cristal, mentira, dormir, sentado, hora, acercarse, bajó, sí, juntos, volvió, peligroso, llevo, leve, cena, caer, corriendo, dices, esperaba, lanzó, lejos, gracias, quieres, volver, hablar, encontró, casi, perro, nadie, nada, qué, suena, contigo, luz, irse, cama, brazo, suelo, dedos, pasos, dispuesta, vivir, entonces, empezaba, cabeza, sabes, garganta, llevaba, miedo, tienda, aliento, era, hombro, repente, moverse, me, estaba, hermana, tono, tienes, ver, quedaba, pareció, vamos, espaldas, esperando, pude, ya, ventana, nariz, gritó, aquello, sacó, subió, dónde, llegué, limpio, empezó, ido, instante, sintió, escapar, seguir, pensamientos, está, piernas, segura, sonrisa, comer, nieve, niña, pequeña, mira, acercó, preguntó, vidas, pensó, dejó, puedes, cerró, verlo, pensando, pasaba, apareció, copa, encima, sentada, mirada, queso, eh, dije, perros, decirle, beso, amigos, abrió, estás, espalda, hacemos, peor, llevado, cara, dolor, estoy, boda, dijo, comida, estar, mano, ojo, dejaba, hielo, plato, sorprendió, cuándo, cerrada, coche, darle, cómo, seguro, cintura, observó, miró, llegar, siente, vaso, camisa, mañana, ansiedad, crees, preguntar, vas, sorprendido, comenzó, descubrir, capaz, madre, tocó, bolsillo, escucha, volví, necesitaba, loco, sonrió, vale, pelo

5 % words correlating most negatively to sentiment:
intereses, consecuencia, corresponden, del, materia, capitales, comercio, relativo, crédito, respecto, pública, militar, municipales, gobierno, estado, fondos, público, imponer, operaciones, exige, militares, impone, capital, relativa, propietarios, mandato, declaraciones, títulos, impuso, enero, anteriores, establecido, propiedad, públicos, diciembre, demanda, gobiernos, junio, marzo, modificaciones, febrero, documentos, corresponde, noviembre, dificultades, abril, manuel, octubre, congreso, legal, autoridades, venta, agosto, vigente, inmediata, comisiones, entrega, ciento, poblaciones, propone, mayo, artículos, presidentes, dominio, presentada, aprobó, fiscal, determina, destinados, generales, cumplimiento, cargos, ruiz, territorio, consideraciones, firmas, civil, vicente, obligaciones, general, civiles, renta, anuales, votos, puerto, fomento, contratos, ministerio, pago,

garantías, mandatario, soberanía, correspondiente, créditos, correspondientes, reformas, citado, considerarse, anterior, costa, comerciantes, ocupa, julio, provincial, párrafo, alvarez, francisco, establece, renuncia, muñoz, garantía, josé, documento, legales, citada, justificar, presidente, derecho, escaso, herrera, manifiesta, ley, establecimiento, extranjeros, título, romero, secretaría, mariano, ortiz, naturales, santiago, república, antonio, sierra, reglamento, efectos, dirigido, aprobado, suprema, año, asegurar, constituyen, establecimientos, fiscales, disposiciones, indispensable, exigir, l, gastos, estableció, presentado, compañías, respectivos, cargo, decreto, antecedentes, acto, razones, poderes, facultad, deberá, minas, recurso, precio, judiciales, distrito, municipal, salas, actos, éste, secretario, hacienda, tránsito, oficiales, superior, provincia, podrán, comprende, funcionario, proyecto, localidad, señalado, pesos, éstos, trabajos, carácter, servicio, atender, necesarios, presente, ministro, deuda, procedentes, junta, tratados, presentar, derechos, clases, riqueza, suponer, segunda, dada, representantes, de, norte, término, carece, refiere, condiciones, éstas, convenio, ejercer, especiales, presupuesto, voto, obliga, ignacio, publicada, defensa, miguel, chile, resolver, tribunal, reserva, jefes, protesta, oficinas, ésta, siguientes, nombramiento, rector, facultades, ejecutivo, materias, diputados, favorable, pri, arias, fecha, actual, campaña, propietario, tarifas, colegios, serán, publicado, importancia, extranjero, dispone, casos

5 % words rising after 1980 and declining before that year:
dificultad, cuidado, dejar, tener, heridas, pasar, conocer, abrir, herida, usar, manera, delante, rival, entendido, reconocer, tiempo, hacer, señales, sucedido, cuánto, cuerpo, pasando, recibir, buena, entrar, placer, mucha, otro, ventaja, malas, haciendo, agradable, mucho, facilidad, sucede, esposo, pidió, tocar, señal, uno, castigo, sitio, salir, deje, entrada, creer, dejan, poco, bajar, inteligencia, ella, subir, confirmó, acudir, pérdida, recorrer, perder, dejado, cerrar, fácil, cuando, sufrir, bien, suelen, sospecha, empezado, buen, parar, visitar, cuerpos, malos, mala, paciencia, persona, negocio, fuera, marido, calor, consejos, lugar, distancia, hijas, muchas, mal, lo, daño, rara, necesitan, siempre, le, hablando, puedo, calma, siguiendo, hijos, sucedió, corta, cortar, objetos, que, hacerlo, sentimientos, con, buenas, negocios, segundos, llevar, acompañado, parto, deseos, miradas, guardar, confianza, suerte, vuelta, reconoció, si, varias, casa, saber, llamar, acababa, justo, silla, quién, veo, padres, tengo, respondió, ven, ponerse, lágrimas, reina, hija, observar, tomar, tiendas, felices, acerca, personas, solo, debajo, completamente, visto, cerca, sencillo, respeto, viendo, lados, puertas, contestó, multitud, modo, probar, necesito, segura, entró, dedo, disfrutar, deja, entender, débil, no, cabeza, faltaba, cuántos, sacó, sorprendido, levantó, deseo, evitar, tranquila, para, les, costado, cuello, cuál, verdad, deseaba, volver, vergüenza, cadáver, salida, porque, príncipe, gusto, horror, enfrente, yo, repente, memoria, abrió, solas, conozco, empezar, permanecer, hacerle, acabó, conocen, estómago, rayo, medicamentos, hijo, pecho, parecer, mi, huesos, terrible, vea, darle, madre, sencilla, perfecto, tuviera, dudas, colocar, desgracia, tomó, pasó, aunque, cuesta, mancha, salido, mejora, vestidos, raro, necesita, cualidades, toque, añadir, debilidad, suele, músculos, llave, talento, víctimas, esperando, respetar, poner, oculta, pensamientos, conoce, igual, creen, malo, hice, pocas, hermano, constantemente, idea

5 % words rising before 1980 and declining after 1980:
industrial, técnicos, existente, norteamericana, industriales, países, fundamentalmente, norteamericano, técnica, constituye, organismos, totalidad, significación, burguesía, plenamente, producción, concretamente, técnico, estrictamente, norteamericanos, período, extranjeras, surge, exterior, reservas, concreta, afirmación, dirigentes, concretas, zona, industria, etapa, expansión, agrícolas, económica, precios, simultáneamente, tiende, cifra, relieve, síntesis, exportaciones, concepción, agrícola, popular, central, banca, campesinos, económico, vigencia, afirmar, posibilidades, aporte, estudio, capitalismo, nacional, períodos, país, orientación, expresó, balance, universitaria, fines, industrias, financieras, inicia, concretos, sectores, desarrollo, existentes, permanente, explotación, obtenidos, limitaciones, tesis, evolución, zonas, científico, subrayó, cifras, organismo, centros, informa, exigencias, personalidades, liberación, base, sindicatos, situación, instituto, problemas, plena, internacional, estructura, lucha, acumulación, inflación, señalar, angel, monetaria, revolución, actitud, conclusiones, venezolano, caracas, excepcional, magnitud, sindicato, dirigente, fundamental, estudia, tendencia, utilización, revolucionario, p, resumen, actuales, sindicales, decisivo, total, inversiones, colaboración, vocación, anteriormente, etapas, ocupación, soluciones, venezuela, definitiva, alcanzado, científica,

transportes, consumo, ingeniería, régimen, financiera, ampliación, planificación, económicas, sector, alcanza, transcurso, histórico, informaciones, interés, regional, socialista, nacionales, realización, aproximadamente, precisamente, oposición, organizado, trabajadores, crisis, ahorro, creciente, privada, transporte, salarios, importancia, ampliar, comité, sindical, política, esquema, perspectivas, iniciado, programación, amplia, actual, oscar, renovación, tasas, américa, solidaridad, estima, incremento, extranjera, dólar, ideología, afán, efectuar, integrado, cabe, constituyen, tarea, intercambio, histórica, cooperación, oficialmente, productos, prestigio, redacción, honduras, universitarios, obrero, efectiva, petróleo, confederación, aniversario, huelga, tendencias, exportación, internas, investigaciones, ramón, economía, económicos, socialistas, dólares, ingreso, elaborado, pagos, fondo, centrales, crecimiento, cuadros, presiones, internacionales, nicaragua, básicos, equivalente, fundamentales, obreros, organización, área, díaz, funcionarios, regionales, avance, reunión, conferencia, populares, brasileño, cultivos, comercial, recientemente, exigencia, comerciales, especialistas, problema, productividad, interno, atlántico, restantes, productores, asistencia, anual, rafael

English Fiction

5 % words scoring highest on first PC axis (PC1):

by, account, present, devoted, such, regard, among, purpose, of, which, received, opinion, expense, whom, shall, advise, effect, upon, difficulty, dignity, quarrel, custom, cease, youth, mere, whose, may, express, great, necessary, nor, distinguished, success, passage, ought, influence, prevent, manner, till, submit, highest, condition, receive, ill, ordinary, daily, altogether, common, proper, delay, absence, interest, generally, engaged, profession, country, possession, its, thus, conduct, consent, evidently, conscience, affected, lend, quarter, advance, declare, aid, wit, plain, afterwards, liberty, principal, extraordinary, marked, speech, remarkable, privilege, letter, fail, behalf, bore, bound, fellow, succeed, duty, subject, propose, sympathy, justice, kindly, superior, these, consideration, peculiar, ignorant, somewhat, fame, occasion, genius, wise, faithful, greater, valuable, indeed, accomplished, possessed, measure, unnecessary, acquaintance, character, fond, disgrace, excellent, vain, sacrifice, obliged, possess, satisfaction, sum, ambition, poor, servant, breach, fare, spirit, powers, permit, alike, rank, extreme, preserve, wealth, humble, splendid, exception, sacred, delightful, reward, certain, dreadful, pains, charge, extend, offence, sickness, forth, behold, sustained, grave, native, importance, proved, interfere, petty, respect, worthy, observe, satisfy, happiness, fair, scheme, gracious, com, mankind, weakness, conviction, bearing, oath, strongly, manners, nature, vast, order, expedition, o, unfortunate, fixed, suffering, positively, voyage, gentleman, unhappy, worship, confess, pity, humanity, instance, praise, courage, habit, fortunate, beauty, congratulate, grounds, objection, dear, noble, savage, broad, require, appearance, intention, pardon, whether, less, landlord, religion, expenses, fatal, existence, stamp, impulse, respectable, maiden, conclusion, departure, false, themselves, fashion, farewell, pleasant, mighty, divide, fortune, thousand, suffer, thou, delight, peace, drawn, defence, demand, entirely, suspicion, painter, shown, withdraw, sorrow, thy, glory, unknown, means, carried, motive, crown, art, thee, advanced

5 % words scoring lowest on first PC axis (PC1):

picked, moving, worked, anyway, upset, empty, stayed, nightmare, tiny, crazy, older, climbing, edge, bother, pick, anyone, finally, banging, shiny, handle, sticky, cooking, across, sweat, outside, crawl, worry, talking, split, somewhere, car, start, smell, about, lot, stopped, biggest, backing, decided, mirror, desperately, cream, kid, upside, restaurant, driving, bowl, tried, telling, damn, stuck, desk, top, waiting, bedroom, floor, overnight, working, halfway, nasty, staring, hell, frozen, blanket, toward, get, woke, driver, leg, slightly, cracked, swim, trying, somehow, lined, wearing, wet, explain, happening, finished, pool, climb, skirt, glasses, worried, racing, birthday, drive, risky, killing, past, lunch, eating, crack, running, happen, chicken, cat, stuffed, try, twisted, belt, tonight, pad, confusing, jump, stays, kitchen, dump, behind, like, cover, stuff, smelled, watching, run, ceiling, switched, handful, watched, siren, nose, catch, coffee, right, tray, wheel, mostly, someone, yelling, crawling, lobby, going, drawer, hooked, lucky, listening, told, skull, baby, trip, getting, scared, poster, package, corridor, belly, pounding, tied, backed, spent, alive, strip, shelf, facing, something, out, tip, spit, relaxed, crash, tomorrow, want, back, sticking, teeth, tomato, everyone, scrambled, hello, freeze, move, intact, carefully, underwear, scare, fist, argue, obviously, twin, lounge, boring, easier, pajamas, wake, slowly,

hungry, discuss, cracking, card, football, black, dug, tasty, busted, whipped, tricky, loose, guess, wall, dig, off, spaghetti, talk, pink, spoon, kill, hit, nowhere, trapped, expensive, embarrassed, elevator, homecoming, leather, embarrassing, despite, towel, skinny, screaming, clothes, switch, nice, timer, tie, bedtime, dizzy, kicking, studied, stretch, blaming, blocked, hanging, up, counter, tension, disappear, slam, anywhere, slap, chips, knife, concentrate, realize, incredibly, adjust, shoot, eventually, scratch, washed

5 % words scoring highest on surging PC axis (PC2):
fallen, as, trusting, promise, spot, friend, hope, soon, fit, safely, deserved, have, forgiveness, disappointment, apology, ever, brother, choose, welcome, so, deserve, spare, owe, tone, fear, distance, much, rising, ease, weak, alarm, might, fault, beautiful, place, heart, nearly, happy, escape, approaching, surrounded, regret, allow, mine, conversation, sweet, prey, reception, fresh, perfect, visit, pause, believing, returned, truth, best, give, truly, strong, eye, myself, forgive, ruined, sad, precious, gig, name, broken, innocent, gentle, hesitate, my, resist, follow, wished, loving, done, throwing, curse, taste, disappointed, purse, interrupting, painful, happier, harm, than, thoughtful, besides, grace, us, sword, with, sister, though, shrink, invitation, feared, daughter, impress, saved, lie, opposite, comfort, compliment, pale, lesson, revenge, and, torture, calm, wounds, cheering, pleased, finding, willing, excited, foot, entrance, touching, desperate, handsome, love, ear, speak, shade, promising, selfish, your, return, introduce, some, storm, proud, sooner, assured, coach, ground, wishing, thankful, led, shelter, more, delicious, sight, stain, sides, low, confusion, secret, gorgeous, kneel, remove, lighten, celebrity, considering, care, housekeeper, groom, being, jealous, meet, mistake, bright, ally, tear, slight, full, strength, eager, supposed, frame, dare, likely, resting, disguise, treat, acknowledge, expression, swear, good, rest, own, draw, excuse, cheat, blow, emotion, grief, deadly, stole, every, seen, interrupt, seated, fully, wander, disappoint, woo, worst, rude, insult, creature, flush, direction, embrace, foul, deny, leave, burst, mansion, towards, dress, who, costume, spoken, patience, wig, hour, shame, complain, found, afford, melody, better, connect, distant, usual, easily, cheer, worn, far, shine, will, offended, angel, last, this, perfectly, pleasure, cast, worse, ready, injured, betray, presence, fortunately, to, fitting, wish

5 % words scoring lowest on surging PC axis (PC2):
material, executive, failure, funds, effective, basis, operate, standard, presentation, organization, technical, treatment, record, development, transportation, procedure, scope, addition, maximum, modern, national, central, vital, scientific, chairman, income, administration, approximately, minimum, indicate, factor, attitude, study, total, criminal, civilization, operation, organized, statement, permanent, tragic, division, association, individual, method, limit, department, federal, social, outstanding, operating, quality, commission, discussion, section, wrote, average, historical, article, attorney, included, conflict, formula, sentimental, ethics, civil, welfare, literature, amateur, production, provide, advertising, publicity, additional, representative, automobile, savings, folk, personality, minor, bureau, motor, loan, western, phrase, cooperation, conference, service, tragedy, district, determine, employment, dramatic, special, labor, local, draft, united, phase, budget, handling, reasonable, century, ideal, tax, actual, employer, evolution, year, identification, inevitable, probation, factory, headquarters, jewish, journal, trial, nowadays, politics, insurance, appeal, generation, policeman, new, offense, function, develop, investment, classified, state, financial, creation, include, director, during, president, responsibility, industry, community, auto, code, rabbi, tremendous, campaign, atmosphere, growth, recent, plant, annual, x, tradition, oil, objective, secretary, technique, program, orchestra, political, writing, evidence, analysis, san, review, maintenance, type, issue, gasoline, law, operator, policy, element, democracy, merely, anyhow, nigger, number, violation, nickel, official, definite, training, buffalo, parole, psychological, represent, personnel, legal, library, written, theory, complete, farm, population, collection, comedy, defendant, worker, editor, construction, professor, sales, chauffeur, equipment, cattle, throughout, telephone, theme, establish, electric, approval, psychology, qualified, prior, ghetto, negro, transferred, enforcement, agency, cow, accused, circuit, comic, solution, item, page, write, suggestion, activity, marvelous, atlantic, example, intelligent, congressman, b, calendar, term, based, symbol, pacific, transfer, action, manual, supreme, vice, poem, period, tales, eliminate, member, title

5 % words correlating most positively to sentiment:
lost, hear, wear, while, his, when, never, lose, deep, fast, let, turning, make, now, take, loud, pair, how, light, looks, but, forget, path, if, seen, believe, hand, arms, taking, surprise, enough, walk, find, breaking, rest, shake, knowing, left, to, seat, toss, sofa, her, rushing, threw, surprised, raised, long, calling, throw, leaving, blood, hoped, pointing, laughing, melt, worse, gave, covered, piece, hang, few, look, set, step, him, here, i, break, gather, bring, arm, struggling, falling, cheek, suspected, comfortable, forward, at, mind, think, together, me, no, call, leave, angry, thanks, stairs, enjoy, burning, begging, blame, couch, dressed, wanting, ride, awake, stepping, breathe, sending, head, grateful, remind, looking, pillow, filled, alone, morning, quiet, close, hold, better, tongue, fill, reach, pain, breeze, ready, catching, instead, toast, smile, easy, turn, distracted, tasted, stopping, whisper, sure, feeling, keeping, next, seeing, forehead, chose, keep, relieved, them, would, tell, sink, beneath, confident, footsteps, air, into, knee, dessert, lead, once, took, moment, inviting, enjoying, ask, one, thank, invite, lip, favorite, breakfast, cup, dangerous, still, kissing, join, steal, gently, shower, congratulations, familiar, breath, with, pulse, memory, safe, what, rescue, side, win, pace, thought, that, learn, shaking, dropping, stand, kiss, pretend, terrified, tear, losing, scumbag, sneaking, waking, cool, wound, then, convince, mother, excuse, burn, whatever, girlfriend, led, kept, you, met, pizza, ringing, limo, bottom, soft, father, knocking, ear, fade, heal, confused, could, least, a, worst, uncomfortable, glow, bleeding, pressing, neck, wrapped, guy, herself, door, your, resting, boot, scary, headed, warm, yourself, flirting, lap, hers, she, database, open, ball, please, sting, not

5 % words correlating most negatively to sentiment:
result, public, rate, reference, title, increase, conviction, established, shown, annual, chief, interest, value, per, provided, direct, capacity, importance, justice, certain, permit, printed, instance, original, institution, rule, article, effect, general, capital, necessary, various, expenses, appeal, p, practical, representative, citizen, addition, acts, reasonable, consideration, society, period, march, establish, commission, profit, complaint, court, settlement, july, cost, act, order, application, examination, authority, state, feature, customs, apply, active, represent, salary, additional, written, committee, demand, scheme, elsewhere, fund, volume, judge, nevertheless, method, secretary, june, january, verdict, jurisdiction, amount, express, land, due, belief, april, growth, penalty, conclusion, authorized, following, religious, supply, during, union, refer, government, governor, independent, stamp, privilege, by, reserve, february, absurd, grounds, division, powers, profession, cent, under, report, chairman, violation, may, proper, f, greater, purchase, request, service, behalf, smith, vote, advance, obligation, county, qualified, moral, applied, board, payment, testimony, its, of, modern, branch, term, judgment, congress, average, unnecessary, among, submit, commissioner, civil, com, august, commonwealth, action, devoted, goods, adopted, extension, council, ought, exception, principal, population, sub, purpose, receipt, such, united, error, maintain, passage, aid, foreign, gross, c, october, coal, president, existence, maintenance, vice, construction, present, extend, december, require, further, opposed, trade, advise, consent, railroad, ordinary, superior, splendid, district, account, third, employment, regard, liable, expense, daily, appointment, sec, relative, spite, custom, extraordinary, generally, stock, these, inspection, burden, transferred, success, strictly, opinion, price, sum, form, constitution, labor, musical, income, serve, policy, session, mill, marked, country, mankind, proposition, shall, subject, immediate, revolution, funds, section, chap, merely, youth, speaker, age, year, september, element, common, speech, clerk, benefit, valuable, mines

5 % words rising after 1980 and declining before that year:
every, selfish, rude, heart, honest, laid, comfort, lately, hesitate, secure, gorgeous, perfect, regret, hope, promise, ruin, connected, spot, sooner, as, thankful, place, danger, besides, compliment, trusting, deserved, nearly, disappointment, friend, apology, truth, owe, prove, mine, glorious, should, same, than, tone, amongst, speak, fit, precious, thrown, alarm, happier, ever, sent, being, save, weak, draw, prey, so, fallen, cheer, dare, truly, happy, grace, ear, distance, willing, sweet, best, approaching, eye, without, safely, opposite, welcome, might, taken, deserve, brave, entering, yet, desperate, attached, injured, disappoint, calm, soon, patience, advantage, done, hearts, engagement, hour, proud, conversation, laying, be, fresh, fault, give, meet, true, brother, mercy, beautiful, lighten, follow, wreck, none, impress, saved, spoken, connect, and, much, strength, excited, have, slight, gentle, resting, sad,

taste, pause, curiosity, will, my, ease, reception, surrounded, stranger, wish, spare, strong, ground, recover, crush, purse, forgive, passing, disappointed, believing, cheering, doubt, any, opportunity, niece, fancy, care, grief, interrupting, return, fear, introduce, wicked, sister, painful, broken, ready, anxious, instant, returned, rising, sight, forgiveness, am, shame, throwing, attend, betray, ladies, entrance, giving, ruined, choose, farther, disturb, treat, trust, kindness, confirmed, rash, insult, allow, lesson, your, presence, with, height, mansion, cheers, satisfied, pass, happiest, usual, delicious, retreat, bless, lord, forth, generous, pray, worst, embrace, deliver, chamber, cannot, throne, distracted, ahem, safe, tear, shrink, gig, us, to, myself, perfectly, cast, remove, kneel, goodness, determined, assured, though, side, curious, eager, sting, offended, cheek, hint, meantime, blessing, blessed, torture, acknowledge, considering, escape, rest, stole, fully, invitation, our, gratitude, curse, wounds, bid, mistake, sunshine, finding, day, mortal

5 % words rising before 1980 and declining after 1980:
basic, function, technique, role, identification, objective, psychological, concept, sequence, program, develop, approximately, sophisticated, typical, technical, relevant, analysis, personnel, breakdown, marvelous, factor, initial, study, percent, basis, traditional, included, presentation, unique, classified, available, isolated, ironic, significant, formula, fiction, personality, based, handling, material, enforcement, soviet, novel, complex, development, million, lack, phase, film, psychiatrist, definition, background, effective, magazine, indicate, director, social, failure, minor, status, psychology, include, tragic, minimum, achieve, suggest, symbol, pattern, central, wrote, tradition, formal, police, local, sample, operating, naive, survival, listed, nuclear, jewish, quality, maximum, environment, cooperation, code, data, amateur, comic, folk, specific, ritual, fantasy, creative, outstanding, review, producer, communist, illusion, billion, impact, policeman, draft, telephone, standard, responsibility, logical, record, ultimate, research, provide, vital, discussion, specifically, ghetto, activity, employee, evolution, budget, reliable, historical, structure, dramatic, x, century, tales, experience, bicycle, cycle, writing, treatment, total, income, procedure, scope, theft, rabbi, federal, publicity, attitude, eliminate, episode, recent, agency, coverage, literature, beginning, organization, operator, executive, criminal, modern, headquarters, civilian, plaza, documentary, funds, addition, conflict, detail, individual, phrase, theme, responsible, scientific, generation, scholarship, laboratory, department, technology, frequency, congressman, organized, businessman, operate, administration, university, particularly, graduate, polish, theater, automatic, critical, concentration, production, tax, myth, chairman, editor, type, culture, legend, final, solution, version, international, equipment, print, attorney, factory, new, section, defense, sales, product, range, dummy, patrol, worker, national, oil, financial, story, understanding, example, civilization, savings, community, article, sexual, sponsor, collection, primary, loan, sentimental, press, begun, tennis, professional, division, physics, training, issue, fascinating, legal, narrator, employer, special, member, journalist, poem, supreme, important, obsession, define, insurance, transportation, representative, motor, reasonable

English excl Fiction

5 % words scoring highest on first PC axis (PC1):
laid, great, useless, evidently, crowded, evening, twelve, sent, prove, very, mere, alike, much, brought, doubt, passing, pass, upon, little, before, broken, hesitate, excuse, hopeless, without, head, shade, plain, wise, brilliant, character, safely, habit, thousand, civilized, returned, so, place, faint, impress, blow, filled, sympathy, regret, nearly, hundred, wit, same, worn, tide, gave, opposite, pale, it, intention, lying, raised, settle, being, bare, bear, bent, hollow, lie, day, profession, lad, town, poison, bore, constantly, man, fellow, executed, perfectly, nothing, worthless, cause, appearance, bound, spoiled, exhausted, motive, owe, possession, shore, last, soon, every, struck, but, delicate, pleasant, lay, against, belong, all, thrown, bark, surely, round, leaving, shining, amusing, ladies, none, sides, cottage, altogether, disturb, burning, whole, horse, he, fancy, now, rubbish, superior, ought, true, moonlight, throw, and, difficulty, sunshine, unfortunate, crooked, helpless, gone, fifty, cast, highest, kindly, pure, cure, say, mouth, honest, farther, maiden, was, fatal, ground, admit, besides, sight, clumsy, ruined, discovered, crown, whatever, nor, attend, worthy, merry, ox, beg, carriage, acquaintance, resting, sum, satisfaction, danger, harmony, twenty, ear, met, proof, seat, no, passage, morning, old, velvet, manner, settled, ready, forty, rotten, not, sooner, to, beneath, seven, drawn, proved, bright, bed, give, boat, magnificent, approaching, neat, maid, forgotten, oath, relieved, reckon,

sailor, starve, declare, satisfied, robbing, handsome, execution, pleasure, ashes, dull, stood, occasionally, beautifully, housekeeper, sudden, master, penny, quarrel, beside, have, after, bow, alone, its, which, house, heads, stand, ever, knight, sheriff, hardly, distance, foolish, rid, wish, admitted, cloth, hand, almost, fourteen, custom, honor, whose, wreck, falling, his, interfere, succeed, supper, cock, sweet

5 % words scoring lowest on first PC axis (PC1):
develop, product, including, scope, presentation, needs, university, use, adult, international, replace, facing, location, testing, directly, technically, specifically, individual, heavily, primary, suggest, split, include, discuss, policy, cycle, between, lethal, background, solve, professional, important, react, scored, involved, nearby, factor, confusing, timer, routine, provide, element, initial, transmission, investment, group, intact, backed, phase, hamburger, status, lack, higher, logical, test, blocked, recognize, effective, nursing, pad, log, possibility, physically, represent, because, travel, involve, physical, pool, alpha, shotgun, zero, problem, drive, b, photo, expensive, technique, active, overnight, incoming, shift, scale, film, activity, sample, biological, low, faced, independent, objective, adjust, current, scientist, decision, assume, leader, zoo, recording, depending, gain, senior, investigating, project, health, typical, difficult, spectacular, winner, panel, specific, financial, stress, formal, naive, replacement, comment, stable, today, blocking, final, study, treatment, ability, quality, determine, chi, development, single, helpful, camping, faster, theater, cigarette, community, compete, listed, g, max, limited, center, frequency, control, occur, function, transfer, student, within, future, investigate, downtown, failure, source, table, locate, normally, concern, research, growth, clearly, closer, campus, medium, contact, range, review, dummy, v, level, definition, identification, older, responsible, enforcement, unfortunately, dramatic, label, kit, trapped, switched, stage, social, ski, biggest, trading, documentary, plot, working, rhythm, breakdown, beach, damage, delta, x, negotiate, setting, available, internal, presidential, recent, used, bus, chips, camera, vehicle, finding, aggressive, employee, flow, structure, promotion, tactical, relationship, concentration, cola, design, card, peak, psychological, taxi, personal, affect, timing, prime, wave, significant, need, market, stack, police, spending, safety, concentrate, economy, encourage, designed, information, delayed, local, bathtub, tend, release, disagree, despite, counter, particular, helping, drugs, regarding, job

5 % words scoring highest on surging PC axis (PC2):
chose, communicate, led, literally, anger, unexpected, compassion, harsh, torture, planet, powerful, argument, sense, peter, essay, confirm, threatening, anxiety, style, breasts, notion, forgiveness, look, confident, celebrity, pursue, memory, intense, expression, angry, scream, precisely, seek, ambush, repeat, dream, universe, discipline, religious, leap, tortured, even, breathing, incredible, fashion, treat, listen, how, capture, often, tom, encounter, invisible, looks, outrageous, brutal, walking, hunger, learn, inspired, limo, godfather, josh, bleeding, pain, gig, heal, breast, suspected, blamed, pill, looking, violence, exercise, like, uncomfortable, flirting, jenny, hidden, stare, greedy, yourself, struggling, introduce, refer, intelligence, imagine, bravo, gay, presence, tiger, luckily, celebrate, demon, popular, suspect, mock, watching, stuffed, birth, walk, sleep, nanny, echo, loser, legitimate, knowledge, comfortable, guilt, joke, allow, disorder, consciousness, tease, woo, magnum, hurt, screaming, swim, unlock, sherry, fade, your, goddess, hell, dragon, arrow, subtle, stalking, slut, language, remind, umbrella, scene, toss, cheating, grounded, shocking, mug, crime, emotion, scandal, eat, healthy, knowing, lied, helmet, choose, examine, potion, wealthy, path, acknowledge, bra, accent, stake, losing, chat, hug, violent, brain, harm, barely, witch, communication, integrity, history, kissing, aware, colleague, feel, spice, hey, follow, hate, sneaking, robin, shower, enjoy, smell, handful, monster, searching, suck, spiritual, stay, distracted, sandwich, universal, remains, rescue, pursuit, wakes, moral, terrified, aha, prey, disco, victim, describe, rap, differently, sally, woke, wedding, stupid, j, miracle, worst, crying, eating, commit, stir, grandmother, manage, song, exciting, belly, scam, female, nap, lean, childhood, attraction, porn, puzzle, convince, fast, you, god, consider, brat, pour, mummy, meant, heather, what, momma, assassination, favorite, tense, instead, madness, puke, expose, add, turns, awkward, magical, talent

5 % words scoring lowest on surging PC axis (PC2):

year, secretary, district, board, july, jersey, assistant, january, president, uniform, june, august, february, month, salary, authorized, station, permanent, april, october, commissioner, west, sec, november, east, accident, meeting, small, supply, south, interest, apparently, state, dealt, vice, branch, september, december, following, covering, belt, inspection, special, statement, crane, steel, fairly, per, less, atlantic, purchase, heavy, under, order, southern, dam, eastern, contract, lease, passenger, hopper, copper, office, commonwealth, northern, transferred, certificate, ash, further, electric, weight, each, other, water, college, exclusive, regular, position, on, paper, pine, sand, engineer, advise, attorney, bull, feed, frankly, operation, charging, strike, except, shipment, slightly, pipe, week, drill, haul, foreman, machine, feeding, drum, goods, actual, in, pig, milk, wet, mines, dry, furniture, cutting, construction, tube, committee, jurisdiction, record, hose, complete, flat, deposit, farm, arrange, warehouse, below, interested, march, general, pacific, announcement, continued, wire, elsewhere, avenue, line, necessary, factory, cent, report, corporation, extension, suggestion, reserve, collected, during, fund, council, price, school, accepted, lake, grade, building, frank, tobacco, officer, pump, experiment, entire, closely, fact, prepared, hospital, north, creek, plant, hatch, amount, ton, expenses, rot, member, warren, barber, reasonable, shipping, various, definite, behalf, resident, agree, association, summer, suction, recently, possibly, gas, connection, hole, dean, gear, dealer, clearing, annual, herd, circuit, finishing, organized, average, greater, tire, handled, pointed, chamber, sheet, importance, roller, fixing, soil, miller, provided, stud, valley, wheat, shop, shorter, grease, nickel, mill, supreme, spite, shallow, clay, club, united, appeal, bureau, poisoning, loaded, for, buffalo, covered, sale, telegram, chairman, dentist, license, demand, rubber, permit, salesman, convention, company, dodge, discharge, carrying, showing, wash, ice, squirrel

5 % words correlating most positively to sentiment:
alive, watch, life, delicious, hair, watched, beating, drove, crowd, knew, lazy, sky, sat, hang, dawn, nights, inviting, hanging, knocking, hide, liar, silly, journey, lived, blew, rang, sit, wait, teach, another, tell, together, body, told, drink, dressed, beat, forgetting, scent, pudding, blame, forget, bad, mother, she, laughing, breath, singing, divide, mystery, sitting, lose, next, shook, seeing, suffering, clothes, laugh, asleep, enjoying, me, suspicious, mad, kiss, couch, breakfast, gather, gossip, find, smiling, smile, polite, steal, when, gently, discover, always, turned, else, my, sometimes, sister, away, wealth, ugly, lonely, ran, whispering, awake, shake, humanity, wore, afraid, invite, ride, endless, saw, drown, draw, perfume, door, understood, devil, calling, dinner, shadow, passionate, face, ancestors, what, poop, forever, worse, friendly, dragged, beyond, thirsty, yell, grew, love, go, thanksgiving, loving, off, intimate, tore, shout, took, breathe, left, down, word, inspiration, embrace, silence, voice, into, her, forgive, turning, hint, smell, loud, dragging, thanks, shaking, crying, conversation, eyes, then, remember, fit, imagination, wander, oh, slept, trusting, babe, dirty, pause, happier, silent, wrong, surprise, drank, naughty, drunk, deadly, father, rob, gorgeous, spit, idiot, moment, barking, spoke, taught, annoying, eager, sunny, fear, boil, wonder, mind, shame, stupid, did, thought, surprised, begging, false, awkward, tasted, embarrassed, wealthy, bread, kissing, tale, suddenly, instance, know, weakness, neck, instead, cheat, passion, song, learned, tent, horrible, dead, lost, knowing, belief, feared, appetite, feeling, laughter, secret, beast, once, adorable, broke, golden, pirate, they, joy, believing, save, cursed, rub, still, horror, beaten, eve, common, spiritual, whoever, reputation, herself, night, sorry, tall, spy, sleepy, stole, forgot, myself, tea, young

5 % words correlating most negatively to sentiment:
manual, surface, marine, chemical, assuming, temperature, technical, determined, rate, base, engineering, tested, direct, library, laboratory, separate, studied, sub, savings, demonstration, request, section, tables, proposal, limit, medical, deputy, limited, envelope, funds, phoenix, foreign, federal, hearing, congressman, draft, identical, size, mentally, storage, indicate, area, fuel, assumed, investigator, isolated, transfer, director, terminal, establish, increase, liquid, determine, strain, chemicals, agreement, p, vacuum, rabbit, transportation, million, central, card, violation, national, assistance, affected, physics, conference, qualified, annual, zone, schedule, underground, generator, rig, employment, addition, prior, ray, result, pressure, administration, automatic, final, program, maximum, training, concentration, tape, procedure, recent, volume, staff, budget, l, shelf, basis, additional, prime, relative, local, included, reaction, rice, finance, established, joint, education, unit, executive, gross, probation, damage, employer, minimum, radiation, equipment, eliminate, rat,

delayed, responsible, vehicle, personnel, operator, medium, current, private, income, scientific, cost, electrical, test, available, explosive, chemistry, concentrate, plus, transmission, effect, range, low, telephone, dollar, producer, reasonable, designed, commercial, control, retarded, application, transport, item, standard, tax, constant, column, graduate, minor, beam, guarantee, cable, notify, clearance, exchange, editor, auto, capacity, oxygen, study, employee, applied, number, blackmail, normally, assist, delta, japan, comment, forest, percent, development, soviet, total, reliable, agency, operating, high, steady, industry, fishing, classified, table, sales, department, coop, psych, apply, peak, maintenance, factor, population, flow, higher, scheme, similar, approval, failure, directly, concerned, occur, capital, breakdown, cigarette, center, scale, effort, permit, field, growth, involved, award, sample, load, retirement, lower, provided, worker, plan, solid, maintain, recording, loan, lab, handling, construction, solution, appreciate, republic, for, investigation, associate, cover, crystal, ford, promotion, formula, represent, backing, pilot

5 % words rising after 1980 and declining before that year:
understood, embrace, perfect, trusting, hearts, believing, seeing, spirit, breathe, footsteps, instead, into, glory, beautiful, desire, comfort, humanity, appetite, joy, forgetting, dead, prayer, flesh, faithful, horrible, saw, suffering, them, wisdom, shine, shadow, instance, endless, knowing, ambition, together, journey, bless, my, truly, place, distracted, her, walk, blessed, breath, awake, promise, mercy, mind, beloved, gorgeous, darkness, imagine, sweet, alive, silence, divide, soul, body, broke, thirsty, myself, voice, me, framed, ashamed, truth, father, draw, never, slept, asleep, patience, disappoint, stole, honest, forget, taught, wander, throw, rushing, calm, themselves, us, inviting, dragged, evil, herself, threw, wandering, heaven, throwing, took, forgot, gather, surprised, hear, perfection, shame, cry, then, steal, loud, faith, happiness, prophecy, away, surprise, blessing, supposed, spiritual, who, silent, loving, fail, brother, feared, forgive, smile, selfish, heavens, glorious, pray, ruined, cheer, accidentally, yourselves, wanting, they, their, beyond, lost, stranger, bread, sat, knew, ourselves, deadly, companion, when, escape, courage, beast, grief, struggling, praying, terrible, horror, every, death, disappointment, mighty, shout, eternal, obey, heal, forgotten, fallen, punish, dress, crowd, once, mysterious, strange, shepherd, beware, sacrifice, heavenly, passion, fit, cheering, she, love, eyes, curse, rest, swore, drew, drink, praise, awful, saved, blame, ruin, whisper, moment, rich, false, learned, mortal, humiliating, thing, jealous, kindness, honesty, let, laugh, dressed, teach, true, watch, calling, hang, eager, laughing, crying, reputation, deserved, suspicious, sword, mad, suffer, sing, word, suspicion, drown, wicked, sorrow, grace, kneel, owe, tasted, hung, harmony, invisible, spoke, rob, belong, eat, deliver, comfortable, sister, apology, surrounded, eye, couch, sit, night, adore, mother, ashes, bible, liar, hide, pretend, misunderstood, thought, son, speak

5 % words rising before 1980 and declining after 1980:
area, program, available, basis, indicate, million, determine, technical, final, development, personnel, unit, tape, replacement, addition, percent, staff, rate, control, plus, eliminate, congressman, equipment, involved, transfer, funds, limited, range, maximum, zone, federal, initial, increase, base, included, responsible, laboratory, budget, tested, procedure, investigator, concentration, administration, result, concerned, effective, minor, savings, separate, chairman, concentrate, prior, nuclear, marine, basic, effort, automatic, peak, maintenance, shelf, designed, tables, test, sample, approximately, current, minimum, system, direct, package, table, hearing, size, chemical, deputy, directly, recent, engineering, manual, chemicals, decision, storage, limit, fuel, b, transportation, billion, representative, data, normally, identical, recording, testing, handling, growth, vacuum, low, schedule, income, level, annual, failure, assuming, worker, review, computer, temperature, department, training, division, trailer, additional, center, terminal, production, national, transport, standard, operating, activity, concern, policy, director, clearance, sophisticated, gross, breakdown, total, conference, retarded, construction, permit, determined, tax, employment, dollar, section, approval, listed, proposal, record, flow, central, radiation, prime, sales, plastic, item, air, underground, primary, isolated, affected, factor, sub, lab, industry, operate, studied, frequency, library, provide, serious, meter, agreement, aircraft, helicopter, physics, electrical, reliable, vehicle, type, unfair, comment, complete, nursing, capacity, pickup, defense, product, request, effect, group, represent, statement, producer, office, nickel, tech, report, loan, medium, employee, operation, special, series, stereo, phase, surface, cost, strip, for, overtime, damage, transmission, pilot, ceiling, executive, occur, lower, auto, major, significant,

problem, supervisor, card, recovery, bureau, clearly, demonstration, identification, cover, rat, analysis, maintain, delayed, volume, similar, senator, emergency, truck, classified, field, draft, higher, ray, investigation, congress, assumed, shallow, extension, since, intact, education, brief, exchange, added, minus, service, population

References

1 Brysbaert, M., Mandera, P., McCormick, S. F. & Keuleers, E. Word prevalence norms for 62,000 English lemmas. *Behavior research methods* **51**, 467-479 (2019).

2 Warriner, A. B., Kuperman, V. & Brysbaert, M. Norms of valence, arousal, and dominance for 13,915 English lemmas. *Behavior research methods* **45**, 1191-1207 (2013).

3 Schmidtke, D. S., Schröder, T., Jacobs, A. M. & Conrad, M. ANGST: Affective norms for German sentiment terms, derived from the affective norms for English words. *Behavior research methods* **46**, 1108-1118 (2014).

4 Redondo, J., Fraga, I., Padrón, I. & Comesaña, M. The Spanish adaptation of ANEW (affective norms for English words). *Behavior research methods* **39**, 600-605 (2007).

5 Glatzeder, B., Han, S. & Pöppel, E. in *Culture and Neural Frames of Cognition and Communication, On Thinking On Thinking* (eds S. Han & E. Pöppel) 233-247 (Springer, 2011).

6 Allen, A. P. & Thomas, K. E. A dual process account of creative thinking. *Creativity Research Journal* **23**, 109-118 (2011).

7 Baas, M., De Dreu, C. K. W. & Nijstad, B. A. A Meta-Analysis of 25 Years of Mood-Creativity Research: Hedonic Tone, Activation, or Regulatory Focus? *Psychological Bulletin* **134**, 779-806 (2008).

8 Morewedge, C. K. & Kahneman, D. Associative processes in intuitive judgment. *Trends in Cognitive Sciences* **14**, 435-440 (2010).

9 Mieda, T., Taku, K. & Oshio, A. Dichotomous thinking and cognitive ability. *Personality and Individual Differences* **169**, 110008.

10 Oshio, A., Mieda, T. & Taku, K. Younger people, and stronger effects of all-or-nothing thoughts on aggression: Moderating effects of age on the relationships between dichotomous thinking and aggression. *Cogent Psychology* **3**, 1244874 (2016).

11 Perkins, J. *Python 3 text processing with NLTK 3 cookbook*. (Packt Publishing Ltd, 2014).
